# Revealing the bacterial diversity and variation of white filamentous microbial mats in marine mangroves of Guadeloupe Island in relation to human activities

**DOI:** 10.1093/femsmc/xtag034

**Published:** 2026-06-16

**Authors:** Mariana Martínez-Noriega, Patrick Jean-Louis, Melody Philippon, Alejandro Sanchez-Flores, Silvina Gonzalez-Rizzo

**Affiliations:** Unidad Universitaria de Secuenciación Masiva y Bioinformática, Instituto de Biotecnología, Universidad Nacional Autónoma de México, CP 62210 Cuernavaca, Morelos, México; Institut de Systématique, Evolution, Biodiversité (ISYEB), Muséum National d’Histoire Naturelle, CNRS, Sorbonne Université, EPHE, Université des Antilles, CP 97110 Pointe-à-Pitre, Guadeloupe, France; Géosciences Montpellier, Université de Montpellier, CNRS, Université des Antilles, CP 97110 Pointe-à-Pitre, Guadeloupe, France; Unidad Universitaria de Secuenciación Masiva y Bioinformática, Instituto de Biotecnología, Universidad Nacional Autónoma de México, CP 62210 Cuernavaca, Morelos, México; Institut de Systématique, Evolution, Biodiversité (ISYEB), Muséum National d’Histoire Naturelle, CNRS, Sorbonne Université, EPHE, Université des Antilles, CP 97110 Pointe-à-Pitre, Guadeloupe, France

**Keywords:** white microbial mats, Beggiatoaceae, mangrove sediments, 16S rRNA metabarcoding, anthropogenic disturbance, environmental metagenomics

## Abstract

White filamentous microbial mats are complex benthic communities, typically structured by sulfur-oxidizing bacteria from the Beggiatoaceae family, yet their diversity and ecological responses in mangrove ecosystems remain poorly characterized. Here, we provide a high-resolution analysis of bacterial communities associated with white microbial mats in marine mangrove sediments of Guadeloupe using 16S rRNA metabarcoding. Bacterial community composition was compared across sites with different levels of anthropogenic impact (protected, natural, and urban). While overall diversity remained stable, richness differed significantly between conditions, and beta diversity analyses revealed clear compositional structuring along the disturbance gradient. A conserved core microbiome was identified across all sites, whereas rare taxa were detected exclusively in urban sites, including *Ferrimicrobium, Thermonospora, Alcanivorax*, and *Serratia*, which has been previously associated with human-induced environmental changes. In contrast, *Prosthecochloris* and *Chlorobaculum* were highly abundant in protected sites, whereas *Sulfurovum* and *Sulfurimonas* dominated urban environments. The relative abundance of Beggiatoaceae also varied across sites, suggesting sensitivity to anthropogenic disturbance. Despite these compositional shifts, measured physicochemical parameters did not significantly correlate with the community structure, suggesting that microbial mat organization is influenced by fine-scale or unmeasured environmental gradients. Together, these findings indicate that white microbial mats respond to anthropogenic disturbance primarily through taxonomic restructuring rather than loss of diversity, highlighting their potential as sensitive indicators of environmental change in mangrove ecosystems.

## Teaser

White microbial mats in mangrove sediments host diverse bacteria whose community composition shifts with human activity, revealing both ecosystem resilience and sensitivity to environmental disturbance.

## Introduction

White microbial mats are visible benthic structures, ranging in thickness from a few millimeters to several centimeters. These mats often consist of filamentous bacterial aggregates arranged over sulfidic sediments, rather than forming the laminated or stratified microbial layers described in microbial mats from other ecosystems, including mangroves (Gerdes 2010, Cadena et al. 2025). They are primarily formed by large sulfur-oxidizing bacteria belonging to the Beggiatoaceae family, which exhibit gliding motility in response to chemical signals (Jörgensen and Gallardo 1999, Dunker et al. 2011). These microbial mats appear as white aggregates due to the accumulation of elemental sulfur (Salman et al. 2013). Filamentous species such as *Beggiatoa* spp. and *Thioploca* spp. typically form mats at the oxygen-sulfide transition zone (Grünke et al. 2011, Salman et al. 2011). They are stabilized by extracellular polymeric substances, including polysaccharides, proteins, and lipids secreted by bacteria (Albuquerque et al. 2010). Moreover, the Beggiatoaceae mats play a crucial ecological role by contributing to primary organic matter production, which supports the development of many other benthic organisms (Buck et al. 2014, Pascal et al. 2014). They also constitute a distinct ecological niche dominated by sulfur-oxidizing bacteria, along with other bacterial groups, as well as eukaryotic and archaeal organisms, but in much lower abundance (Prieto-Barajas et al. 2018).

White microbial mats have been reported to proliferate in a wide range of environments, including freshwater and marine environments, such as lagoons, lakes, hydrothermal vents and cold seeps, highlighting their ecological versatility (Stal 2001, Baumgartner et al. 2006, Teske and Salman 2014). In tropical coastal ecosystems, they often coexist with phototrophic organisms such as cyanobacteria and microalgae (Bernard and Fenchel 1995, Guidi-Rontani et al. 2014). However, their occurrence and community structure in sulfidic coastal environments such as mangroves remain poorly documented. To date, only a few studies have specifically examined these mats in marine mangrove sediments. Two bacterial species associated with these benthic mats, *Candidatus* Maribeggiatoa and *Candidatus* Isobeggiatoa, were identified in mangrove sediments of Guadeloupe island (Jean et al. 2015), marking the first report of the genus *Isobeggiatoa* outside Northern Europe and Arctic waters. Yet the broader bacterial diversity and taxonomic composition of white microbial mats in marine mangroves remain largely unexplored.

Recent metabarcoding studies have demonstrated the value of 16S rRNA gene sequencing for resolving the taxonomic composition of microbial mats across sulfur-rich environments. In sulfur-rich ecosystems, Fray et al. (2024) showed that white filamentous microbial mats developing under low-oxygen, high-sulfur conditions exhibit distinct community structures shaped by local environmental conditions. Likewise, Crépeau et al. (2011) reported high microbial diversity within visually homogeneous microbial mats in hydrothermal vent systems using DNA-based approaches, emphasizing the role of redox gradients and micro-niches in structuring mat-associated communities. Together, these studies provide a broader methodological and ecological framework for interpreting microbial mat diversity and support the use of metabarcoding approaches to explore microbial mat diversity in complex, sulfur-influenced ecosystems such as mangroves (Crépeau et al. 2011, Fray et al. 2024).

Mangrove ecosystems are located at the interface between land and sea, occurring across 60 to 75% of the world’s tropical and subtropical coastlines (K et al. 2024). They provide vital ecological services, such as carbon sequestration, coastal protection, and habitat provision for a wide range of organisms (Asari et al. 2021). However, these ecosystems face increasing pressure from human activities, including urban development, pollution and land-use change (Bouchez et al. 2013, Song et al. 2023). Microbial communities play a critical role in the biogeochemical cycles of mangrove ecosystems, and disruptions caused by human activities can significantly alter their structural and functional diversity (Andreote et al. 2012, Ghose et al. 2024). Assessing the taxonomic composition of white microbial mats, in response to anthropogenic gradients may provide valuable insights into ecosystem health and resilience. To address this, we used a 16S rRNA metabarcoding approach to characterize, for the first time at the molecular level, the bacterial composition of white microbial mats in marine mangrove sediments from Guadeloupe Island (FWI). The aims of this study were to (i) characterize the taxonomic composition of these mats, and (ii) assess how bacterial communities vary across sites exposed to different levels of human disturbance. We hypothesized that increasing anthropogenic influence would be associated with shifts in community composition, particularly the emergence or dominance of taxa associated with disturbed environments. These insights may help evaluate the ecological sensitivity of microbial mats and their potential role as bioindicators in tropical mangrove ecosystems.

## Materials and methods

### Site and sampling

**Figure 1:**
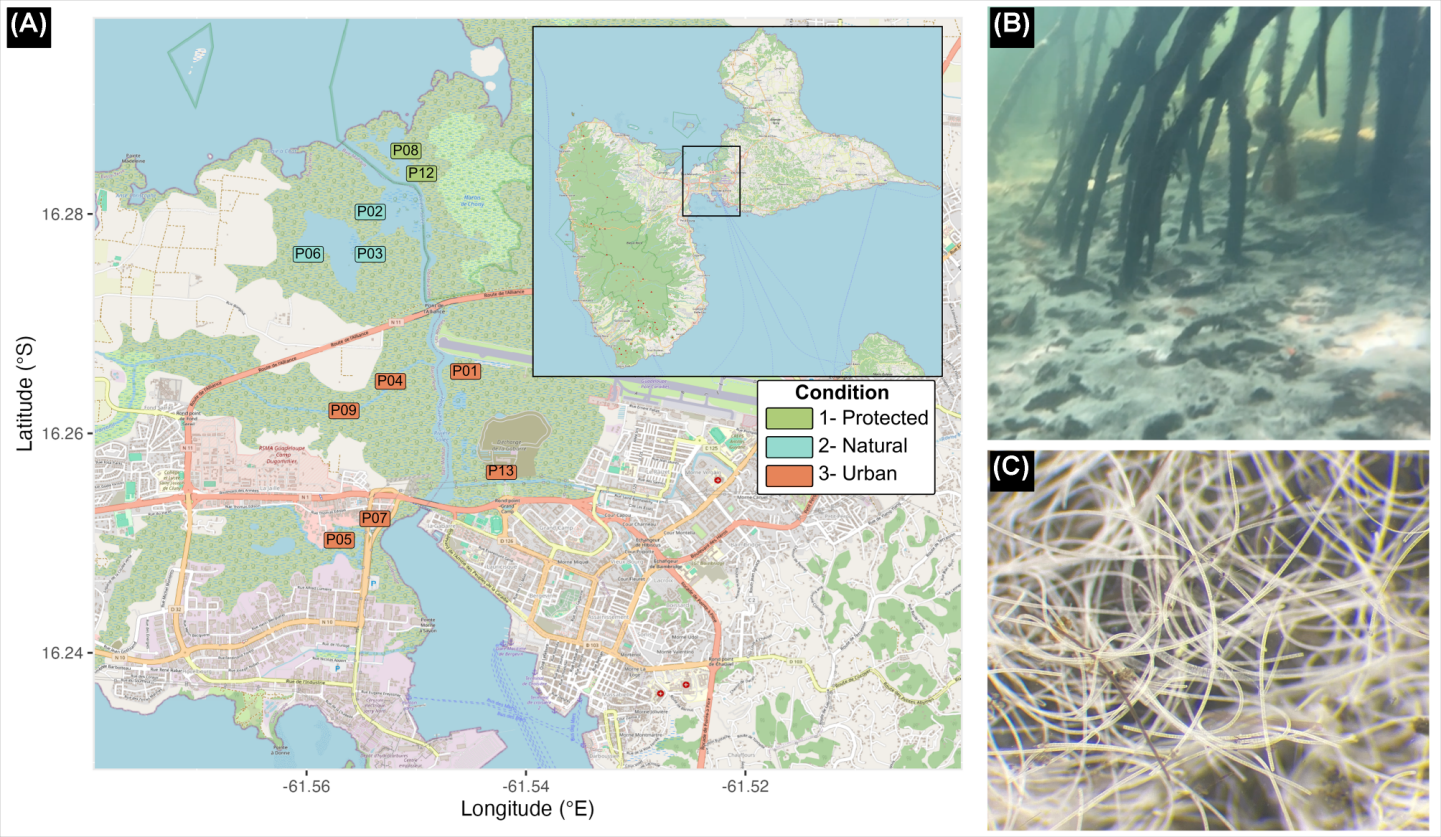
Localization and visualization of white microbial mats across different mangrove conditions on Guadeloupe Island (FWI). (A) Map of sampling sites along the marine channel of the *Rivière-Salée*. Sampling sites were categorized by environmental conditions as follows: Protected mangroves (P08, P12), Natural mangroves (P02, P03, P06), and Urban mangrove sites (P01, P04, P05, P07, P09, P13). All samples were collected from visible white microbial mats. (B) Representative field photograph of white microbial mats covering sediment around *Rhizophora mangle* roots and landscape that characterize the sampling site environments. (C) White microbial mats samples observed under a dissecting microscope.

Sampling was performed during the dry season (February–March 2022) in the marine mangroves of Guadeloupe Island (French West Indies), specifically along the Rivière Salée marine channel separating Basse-Terre and Grande-Terre (Fig. 1A). Eleven sampling sites were selected to represent a gradient of anthropogenic impact, including urbanized, natural and protected areas: (i) urban—sites located in close proximity to Jarry, an industrial zone influenced by human activities such as airport operations, a landfill, and major transportation infrastructure; (ii) natural—sites situated north of the channel, within the Manche à Eau lagoon, and (iii) protected—sites located within the boundaries of Guadeloupe National Park, where strict regulations on navigation, monitoring, and conservation are enforced in accordance with French National Parks guidelines. As no specific chemical pollutants were directly measured in this study, this classification reflects differences in overall anthropogenic influence rather than quantified contaminant levels. Sampling within the protected national park was conducted after obtaining the appropriate permits from the competent environmental authorities. At each site, four replicates were collected, resulting in a total of 42 samples (except sites P04 and P07, where only three samples were obtained). Microbial mats were sampled using a 60 mL sterile syringe by gently aspirating the white filamentous mats present on the sediment surface. The four replicates were spaced approximately 1 m apart to capture fine-scale spatial heterogeneity. Samples were immediately placed on ice in the field and transported to the laboratory, where they were stored at −80°C for subsequent molecular and physicochemical analyses.

### DNA extraction and sequencing processing

Total DNA was extracted and purified from 250 mg of microbial mats using the DNeasy PowerSoil kit (QIAGEN), following the manufacturer’s protocol. The V3-V4 region of the 16S rRNA gene was amplified using S-D-Bact-0341-b-S-17 and S-D-Bact-0785-a-A-21 primers as previously described (Klindworth et al. 2013). Amplicon libraries were prepared according to the standard Illumina protocol and sequenced on the Illumina MiSeq platform with a paired-end configuration of 2 × 300 cycles.

### Bioinformatic processing and taxonomic annotation

Raw paired-end reads were processed to reconstruct the original amplicons (average length ∼463 bp) using Flash v1.2.11 (Magoč and Salzberg 2011). To maximize the high-quality assembly of the V3-V4 region, a maximum overlap of 250 bp (-M) was established, with a maximum mismatch density of 0.6 (-x) to ensure stringent alignment while maintaining a high yield of merged fragments. Only the successfully merged sequences (*extended fragments*) were retained for downstream analysis, while uncombined reads were discarded.

Taxonomic assignment was performed using Parallel-META pipeline v2.4.1 (Su et al. 2014). Sequences were queried against the Metaxa2 database v2.1.1 (Bengtsson-Palme et al. 2015), which provides high resolution for SSU rRNA sequences. The classification was executed with an E-value threshold of 1 × 10^−30^ (-e) to ensure robust statistical significance in the hits, following the workflow previously described in (Escobar-Zepeda et al. 2015).

All raw sequencing data have been deposited in the NCBI Sequence Read Archive under BioProject ID PRJNA1281175.

Abundance matrices were generated from the classification.txt output of Parallel-META across all taxonomic ranks. The genus level matrix was imported into R v3.4.1 to construct a Phyloseq object v1.42.0 (McMurdie and Holmes 2013).

Community ecology analyses, including alpha and beta diversity, were performed using the Vegan v2.6.4 and phyloseq while multivariate visualizations and Principal Component Analysis (PCA) were generated using FactoMineR v2.8 and ggplot2 v3.4.3.

Alpha diversity indices were calculated using the phyloseq package in R, and statistical significance was evaluated using a Kruskal–Wallis test followed by Dunn’s post hoc comparisons. A *P*-value threshold of 0.05 was applied to determine statistical significance. Beta-diversity was assessed using non-metric multidimensional scaling (NMDS) based on a Bray–Curtis distance matrix, computed with the metaMDS function. Differences among groups were tested using permutational analysis of variance (PERMANOVA) with Benjimani–Hochberg correction for multiple comparisons performed with the adonis function from Vegan R package with a 0.05 *P*-value.

To evaluate potential site-level effects, additional PERMANOVA analyses were conducted with permutation constraints using sampling site as a blocking factor. Pairwise PERMANOVA comparisons were performed to estimate effect sizes (R²) among environmental categories.

Taxonomic abundance was visualized with stacked-bar plots generated using tidyverse v2.0.0 (Wickham et al. 2019) and ggplot2 v3.4.3 packages (Wickham et al. 2016). Principal component analysis (PCA) was performed using the dudi.pca function from ade4 v1.7.22 R package (Dray and Dufour 2007).

To assess shared and exclusive genera among site categories (Protected, Urban, and Natural), a Venn diagram was constructed. First, a filtering step was applied to retain only those genera whose cumulative relative abundance across all samples exceeded 1%. This approach aimed to exclude low-abundance or sporadic taxa, focusing the analysis on the most ecologically relevant genera. The filtered abundance matrix was converted into a presence/absence matrix, where a genus was considered present in a sample if its relative abundance exceeded 0.1%. Taxa considered absent in each site were not detected in the sequencing dataset under the applied filtering thresholds and may reflect detection limits of the amplicon-based approach rather than true ecological absence.

Core microbiome analyses were conducted at the genus level, which provided the most consistent and ecologically interpretable taxonomic resolution for this dataset; preliminary evaluations at higher taxonomic ranks revealed inconsistencies related to database-dependent family assignments. The Venn diagram illustrating the overlap and exclusivity of genera among sites categories was generated using the Venn Diagram R package (Chen and Boutros 2022).

### Physicochemical measurements

During the sample collection, a multiparameter probe (HANNA instruments HI9829, Woonsocket RI, USA) was used to measure *in situ* environmental variables, including oxygen concentration, conductivity, temperature, salinity, and dissolved oxygen. Additional physicochemical parameters, such as nitrates, nitrites, ammonium, and organic matter were determined *ex situ*. Nitrite and nitrate concentrations were measured by spectrophotometry following the method described by Schnetger and Lehners (Schnetger and Lehners 2014). In brief, for nitrite measurement, samples were placed in a 96-well microplate with one-tenth volume of Griess-reagent prepared according to a standard protocol. For nitrate determination, an equal volume of sample, Griess-reagent and 1% (w/v) vanadium solution were added to a 96-well microplate. The microplates were incubated at 40°C for 30 min and absorbance was measured at 540 nm. Ammonium was determined using the phenol-hypochlorite method (Solorzano 1969). A 1000 mg/l ammonium stock solution (Merck) was used to prepare 500 µM working standard solution daily. A 1 ml aliquot of each sample was mixed with 40 µl of sodium nitroprusside solution (reagent I) and 40 µl of alkaline solution (reagent II), incubated in the dark for at least 6 hours and the absorbance was measured at 630 nm. To quantify organic matter (OM) sediments samples were (i) oven-dried at 105°C for 48 h to estimate water content and (ii) subsequently heated at 550°C for 2 h in a muffle furnace to determine organic matter content by loss on ignition (LOI).

### Differential abundance analysis

We performed a differential abundance analysis using ALDEx2 v3.22 (Gloor et al. 2016) to statistically identify taxa that differ significantly among environmental conditions using a Kruskal–Wallis test with Benjamini–Hochberg correction and complementary visualizations using a bubble plot and a heatmap using the same package.

## Results

### Sequence analysis and bacterial diversity indices in microbial mats across three distinct mangrove conditions

A total of 42 samples, collected from mangroves under three different environmental conditions (urban, natural, and protected), were sequenced yielding approximately 100 000 reads per sample. On average, 155 274 high-quality paired-end reads were obtained per sample (Supplementary Table S1) and after quality filtering and processing, an average of 152 324 paired end reads were retained, of which 144 941 (97.90% on average) were successfully reconstructed as V3-V4 16S rRNA amplicons. An average taxonomic assignment rate of 94.85% was achieved. Rarefaction curves indicated sufficient sequencing depth to capture most microbial diversity, with low singleton abundance ensuring robust downstream analyses in all samples. (Supplementary Figure S1).

**Figure 2:**
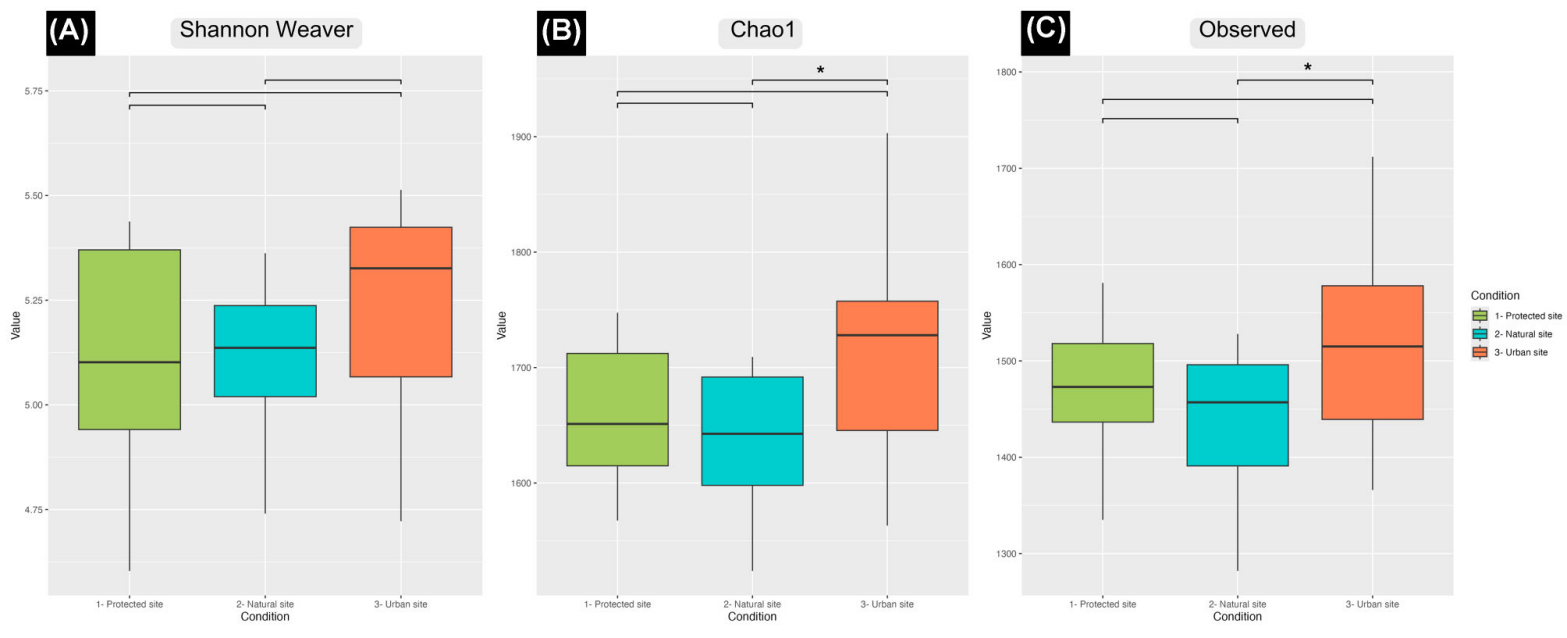
Alpha diversity indices across three mangrove conditions: protected sites (left box), natural sites (middle box), and urban sites (right box). Boxplots display the distribution of Shannon–Weaver, Chao 1, Observed Taxa indices (from left to right). Horizontal lines and asterisks (*) indicate statistically significant differences between conditions (*P* < 0.05) evaluated using a Kruskal–Wallis test followed by Dunn’s post hoc comparisons.

To describe the richness and diversity within microbial mats across the three conditions, alpha diversity indices including Observed Taxa, Chao1, and Shannon–Weaver were calculated (Fig. 2). Alpha diversity varied moderately among samples across environmental conditions. A Kruskal–Wallis test revealed no significant differences in Shannon diversity among environmental conditions (*P* = 0.198). In contrast, richness metrics differed significantly among conditions: both Observed Taxa (*P* = 0.043) and the Chao1 index (*P* = 0.035) showed significant variation. Post hoc Dunn’s tests indicated that these differences were restricted to comparisons between natural and urban sites (Observed Taxa: *P* = 0.033; Chao1: *P* = 0.042).

**Figure 3:**
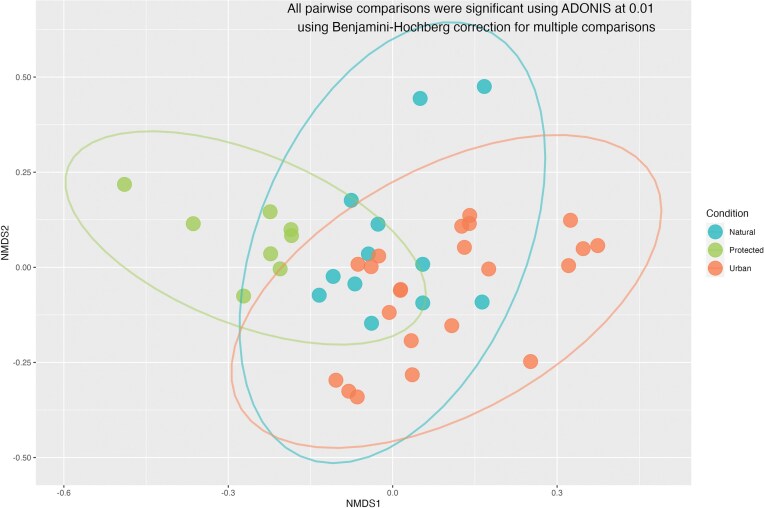
NMDS ordination of microbial community composition across environmental conditions: Each point represents an individual sample, colors indicate site categories: natural (middle ellipse), protected (left ellipse), and urban (right ellipse). Ellipses represent 95% confidence intervals around the centroid of each group. Differences in community composition among sites categories were statistically supported by ADONIS tests, showing significant differences among all pairwise comparisons (*P* < 0.01), with Benjamini–Hochberg correction applied for multiple testing.

To evaluate differences in community composition, non-metric multidimensional scaling (NMDS) based on Bray–Curtis dissimilarities was performed (Fig. 3). This analysis revealed clustering of samples according to environmental condition, with protected sites forming a distinct group, while natural and urban samples showed partial overlap. PERMANOVA analysis confirmed significant differences in community composition among environmental conditions (*P* = 0.001).

To determine whether these patterns could be influenced by within-site variability, homogeneity of multivariate dispersions was assessed using PERMDISP. No significant differences in dispersion were detected among sites (ANOVA, F = 0.423, *P* = 0.924), indicating comparable dispersion across groups. A principal coordinates analysis (PCoA) with 95% confidence intervals per sampling site showed that replicate samples generally clustered by site while still displaying measurable intra-site heterogeneity (Supplementary Figure S2). Within-site variability in physicochemical parameters was further explored using replicate measurements summarized in Supplementary Table S4 and visualized through a site-level PCA (Supplementary Figure S4).

To further evaluate site-level effects on community composition, PERMANOVA analyses were repeated with permutations constrained by site (blocking factor). The global model indicated that environmental condition explained a significant proportion of community variance (R² = 0.173, *P* < 0.05). Pairwise comparisons showed that differences between Urban and Natural sites remained significant (*P* = 0.032; R² = 0.076), whereas the contrast between Urban and Protected sites showed a larger effect size (R² = 0.165; *P* = 0.002).

### Taxonomic composition of bacterial communities in mangrove microbial mats

**Figure 4:**
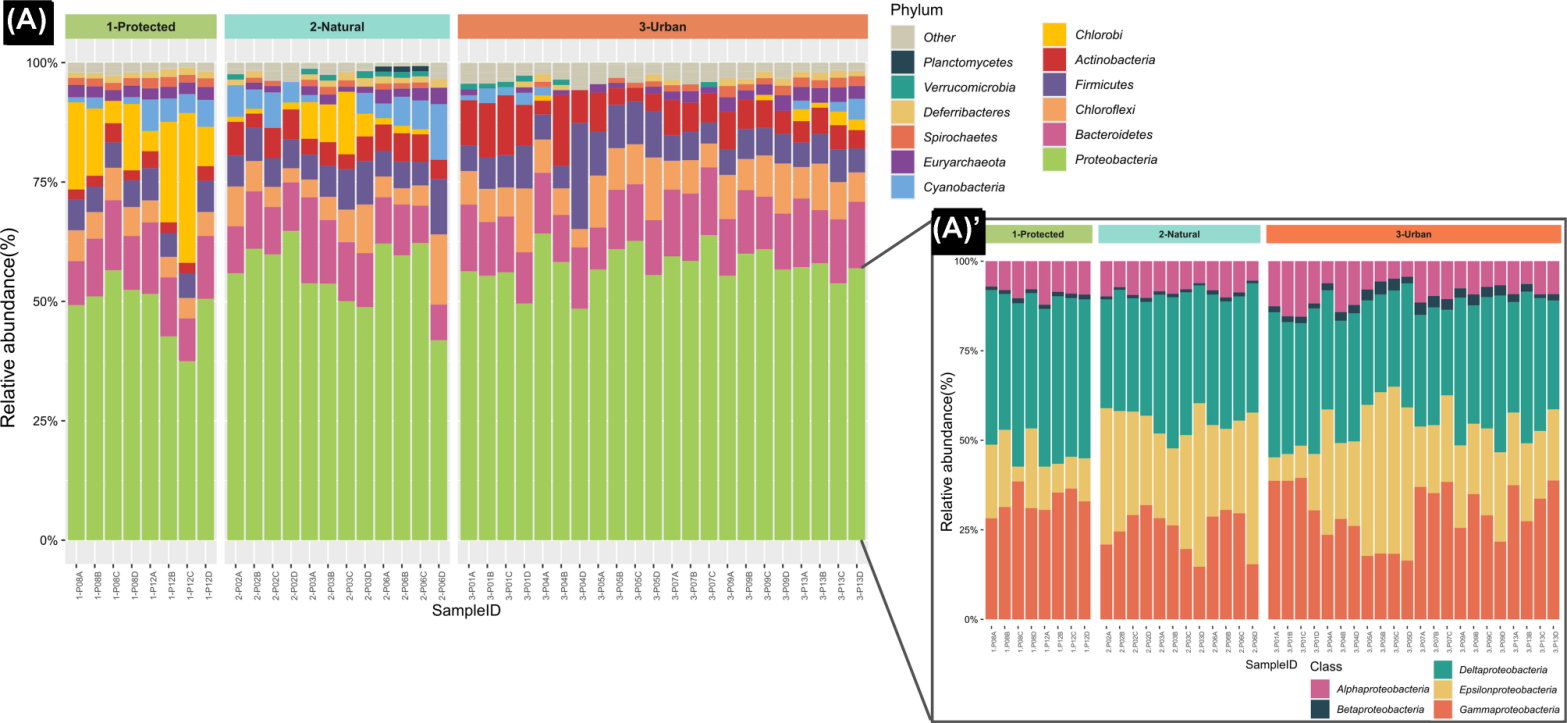
Microbial community composition across environmental conditions, displayed at two taxonomic levels: (A) Relative abundance at the phylum level (A’) Relative abundance at the class level within Proteobacteria. Bar plots represent individual samples from protected, natural, and urban sites. Only the 12 phyla with a relative abundance equal to or greater than 1% are depicted.

The taxonomic composition analysis identified 28 bacterial phyla (98.01% of total sequences) and 3 archaeal phyla (1.99%). Among bacterial taxa, 12 phyla exceeded 1% relative abundance (Fig. 4). Proteobacteria dominated all samples, followed by Chlorobi, Bacteroidetes, Chloroflexi, Firmicutes, Cyanobacteria, and Actinobacteria (Fig. 4A). Relative abundance of several phyla varied across environmental conditions, notably Chlorobi, which decreased along the disturbance gradient from 14.46% in protected sites to 3.17% in natural sites and 1% in urban samples. In contrast, Actinobacteria were more abundant in urban samples (6.7%) than in protected sites (2%). Cyanobacteria also tended to be more abundant in several samples from protected and natural sites than in urban ones (Fig. 4A). Within the phylum Proteobacteria, Deltaproteobacteria, and Gammaproteobacteria were the most abundant classes representing 19.93% and 16.12% of sequences, respectively, whereas Alphaproteobacteria and Betaproteobacteria occurred at lower abundance (Fig. 4A’).

### Differential abundance of bacterial genera across environmental conditions

**Figure 5:**
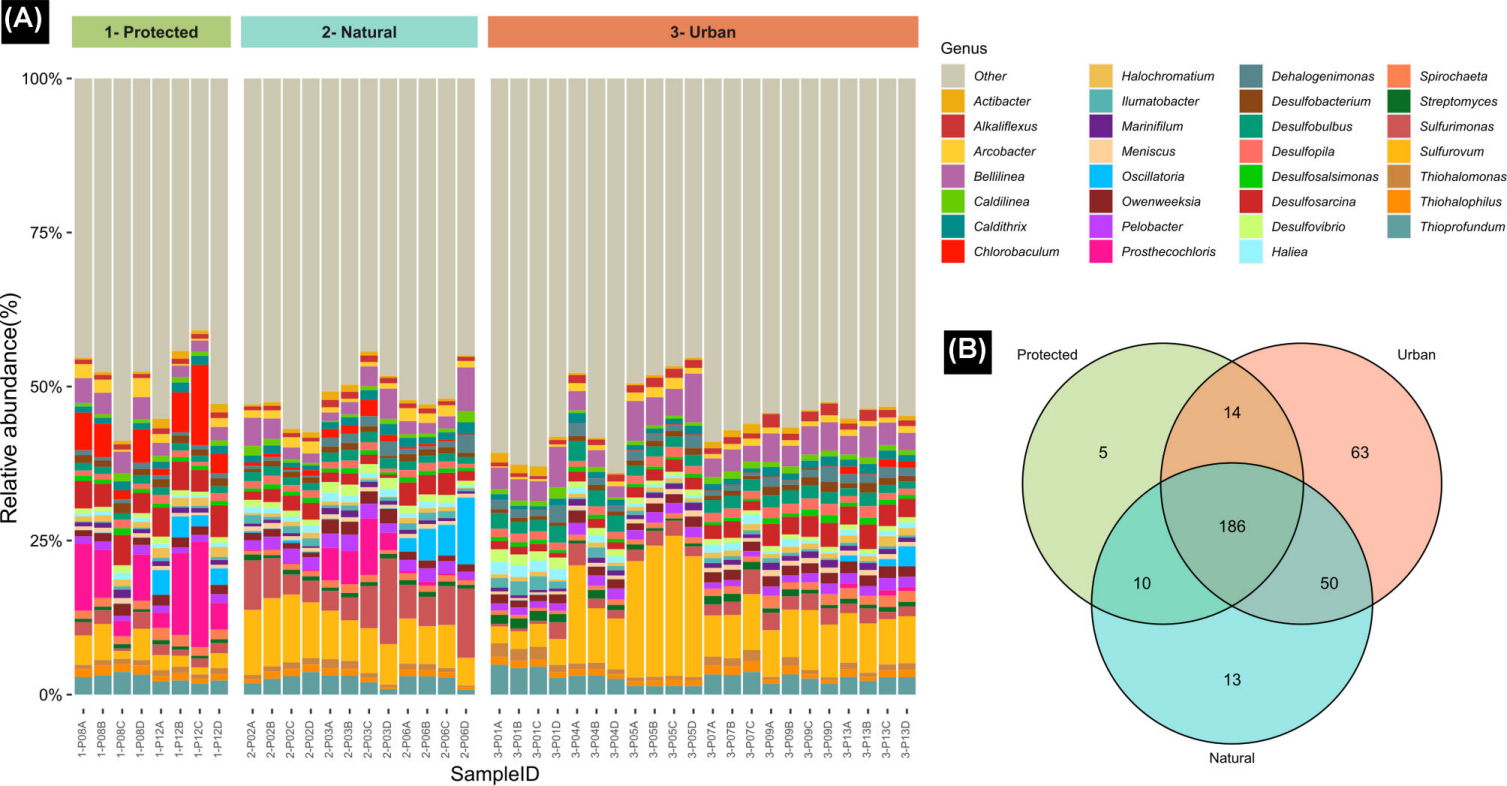
Shifting microbial landscapes: community composition and taxonomic intersections (A) Microbial community composition at the genus level across samples from protected, natural, and urban sites. Each bar represents an individual sample; colors indicate bacterial genera. The “other” category groups low-abundance genera. (B) Distribution of core and unique genera among the three environmental conditions. Numbers indicate genera unique to each condition or shared across two or all three conditions.

Regarding the bacterial composition at the genus level, microbial mats comprised 2103 genera across all samples (Supplementary Table S2), with the 30 most abundant genera (>1% relative abundance) shown in Fig. 5A. Several dominant genera exhibited condition-specific patterns. *Prosthecochloris* spp. was more abundant in the protected sites (8.22%) and detected in the natural sites (2.06%) but was absent from urban samples. Similarly, *Chlorobaculum* spp., was observed only in the protected sites (5.2%) and natural sites (1%).

In contrast, the relative abundance of *Sulfurovum* spp., and *Sulfurimonas* spp. were higher in urban sites (8.98% for both) than in protected sites (3.08% and 1.67%, respectively).

### Core microbiome composition

To characterize the core microbiome across environmental conditions, we identified genera shared among all samples, after applying a relative abundance threshold of 0.1% (Fig. 5B and Supplementary Table S3). This analysis revealed a total of 186 shared genera, representing 63%–85% of the microbial communities. Urban sites exhibited the highest number of unique genera (63), whereas protected sites had the lowest, with only five. Details regarding the common and unique taxa and their abundance among environmental conditions can be found at Supplementary Table S3.

**Figure 6:**
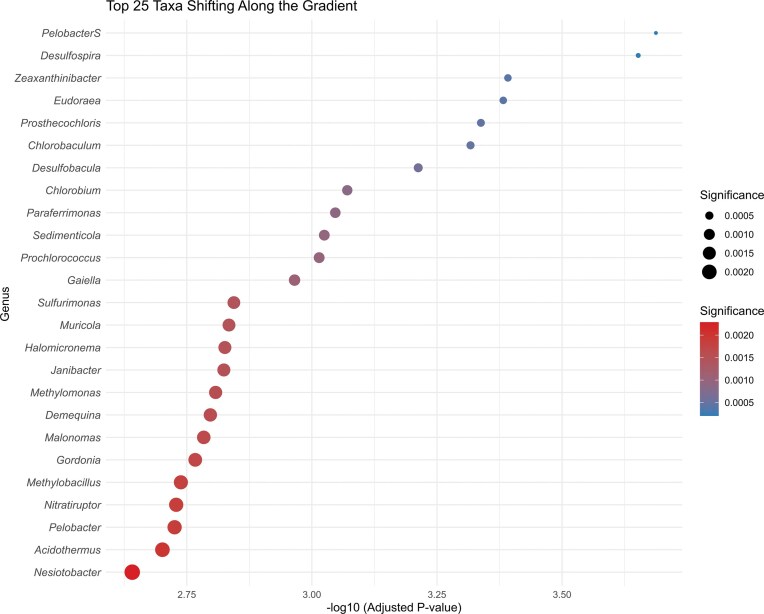
Differentially abundant bacterial genera across mangrove environmental conditions. Bubble plot summarizing the results of the differential abundance analysis performed using ALDEx2 at the genus level. Only genera showing significant differences among protected, natural, and urban sites (Kruskal–Wallis test with Benjamini–Hochberg correction, adjusted *P* < 0.05) are displayed. Bubble size reflects the effect size, while color intensity represents the strength of the statistical signal. This analysis provides statistical support for taxa associated with the environmental gradient across mangrove conditions.

To statistically identify bacterial taxa associated with the different environmental conditions, we performed a differential abundance analysis using ALDEx2 at the genus level. Several taxa showed significant differences in relative abundance among protected, natural, and urban sites (Kruskal–Wallis test with Benjamini–Hochberg correction, adjusted *P* < 0.05). Taxa significantly associated with the urbanization gradient are summarized in a bubble plot highlighting effect size and significance (Fig. 6), while a heatmap representation illustrates consistent shifts in relative abundance patterns across environmental conditions (Supplementary Figure S3). These results provide statistical support for the compositional e patterns observed in taxonomic profiles.

### Beggiatoaceae species diversity in white microbial mats across conditions

**Figure 7:**
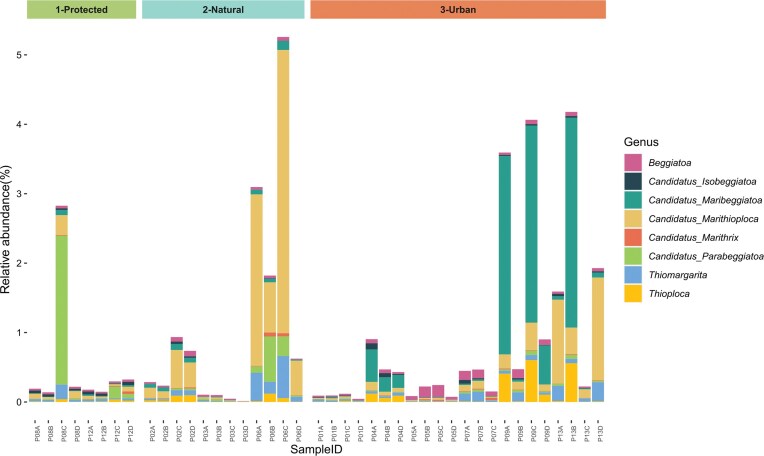
Relative abundance of Beggiatoaceae family members across samples. Abundance is expressed as a percentage of the total microbial community. Sample P06C showed the highest relative abundance, exceeding 5%.

Given their role as the dominant filamentous bacteria forming white microbial mats, members of the Beggiatoaceae family were examined in detail across environmental conditions (Fig. 7). Beggiatoaceae-related genera were detected in all samples, with variable relative abundance across sites. Although overall Beggiatoaceae abundance was low in the protected sites, *Ca*. Parabeggiatoa reached 2.4% in a single sample (P08C). In natural sites, *Ca*. Marithioploca was the most abundant lineage, reaching up to 4.07%, whereas urban samples were characterized by predominance of *Ca*. Maribeggiatoa, with values up to 3.02%. *Ca*. Marithioploca was also detected in urban samples at lower abundance (0.2%). Interestingly, the highest overall abundance of Beggiatoaceae-related genera was observed in natural samples, reaching 5% in one sample.

### Physicochemical landscape and biodiversity patterns

A Principal Component Analysis (PCA) was conducted based on metadata obtained from a multiparametric probe and subsequent laboratory analyses (Supplementary Table S4). The aim of this analysis was to explore how physicochemical variables vary across sampling sites and environmental conditions.

Although three distinct community profiles were previously identified in the beta-diversity plot (Fig. 3), the PCA did not reveal a clear separation of samples based on the predefined environmental conditions, and they did not correlate with the measured physicochemical parameters (Supplementary Tables). Instead, samples tended to cluster more tightly within individual sites rather than between condition categories, suggesting that local environmental variability plays a stronger role in shaping physicochemical profiles than the broader classification of sites by disturbance level. Environmental variables such as conductivity, organic matter content, nitrates, nitrites, dissolved oxygen concentration and percentage were associated with several samples mainly from natural sites. Variables such as temperature, pH, and ammonia were more closely aligned with samples subset of urban sites. The rest of the samples are scattered with no physicochemical parameter strongly associated. To further examine physicochemical variability at a finer spatial scale, a site-level PCA was performed using the same set of environmental variables. This analysis highlights heterogeneity among sampling sites, including within the same environmental category, and is provided as Supplementary Figure S4.

## Discussion

### Diversity patterns and environmental structuring of microbial mats

Our study provides the first detailed characterization of the bacterial communities associated with white microbial mats in marine mangrove sediments. These mats host diverse bacterial assemblages whose composition varied significantly across environmental conditions, indicating that these communities are structured by local ecological filtering along the disturbance gradient. Although Shannon diversity remained relatively stable among environmental conditions, richness metrics differed significantly between natural and urban sites, suggesting that disturbance influences taxonomic richness more strongly than community evenness. The broader diversity observed in urban sites is consistent with patterns reported in other disturbed mangrove and coastal ecosystems, where anthropogenic impact can increase microbial diversity and promote the emergence of rare taxa. This highlights the ecological sensitivity of low-abundance microbial community members to environmental changes (Santos Dos et al. 2011, Maiti and Chowdhury 2013, Li et al. 2019, Ghose et al. 2024).

Protected sites in contrast, exhibited a more cohesive community structure, suggesting relatively stable and distinct microbial assemblages, whereas natural and urban sites showed partial overlap. This overlap may reflect intermediate disturbance or unquantified environmental pressures, such as pollutant influx through hydrological connectivity, leading to similarities between natural and urban sites. Previous studies have shown that microbial communities respond not only to current abiotic parameters but also to historical land use and chronic disturbance (Allison and Martiny 2008, Nemergut et al. 2013).

Similarly, PCA analysis revealed site-specific variability but no clear separation according to disturbance level and measured physicochemical parameters did not correlate strongly with taxonomic profiles. However, multivariate analyses indicated that community composition remained significantly structured across environmental conditions even after accounting for site-level variability, while no difference in multivariate dispersion were detected among sites. Together, these results suggest that the microbial community composition is shaped by environmental factors, not fully captured by bulk physicochemical measurements, potentially including micro-scale geochemical heterogeneity, unmeasured stressors, or cumulative disturbance histories. Similar decoupling between measured environmental variables and microbial community composition has been reported in other benthic ecosystems, reinforcing the potential of microbial mats as sensitive indicators of both present conditions and cumulative ecological pressures (Preisner et al. 2016, Mazière et al. 2023).

The taxonomic composition of white microbial mats, dominated by Proteobacteria, Chlorobi, Bacteroidetes, Chloroflexi, and Cyanobacteria, is consistent with phyla commonly reported in sulfidic environments (Schulz and Jørgensen 2001, Mußmann et al. 2003, Luis et al. 2019). In particular, the prevalence of Proteobacteria, especially Gammaproteobacteria and Deltaproteobacteria, aligns with the prevalence of microbial groups involved in sulfur cycling and organic matter degradation in a marine and coastal systems (Aranda et al. 2015, Alzubaidy et al. 2016, Ullah et al. 2017, Zhou et al. 2017, Quintero et al. 2022, Gómez-Acata et al. 2023). Together, these results support the interpretation that white microbial mats in mangrove sediments constitute structured sulfur-associated microbial consortia. However, functional inferences at this taxonomic resolution should be interpreted with caution, as metabolic capabilities cannot be directly inferred from 16S rRNA gene data alone.

### Core microbiome and taxonomic shifts associated with urban disturbance

Despite the taxonomic variation observed across environmental conditions, a relatively stable core microbiome was identified in white microbial mats of Guadeloupe, suggesting that these communities retain a conserved taxonomic backbone across mangrove conditions. Many taxa within this core microbiome belong to bacterial phyla commonly associated with microbial mat ecosystems including Proteobacteria, Chlorobi, Bacteroidetes, Chloroflexi, and Cyanobacteria.

Several core taxa identified in this study have previously been associated with essential metabolic processes in aquatic and sediments environments including organic matter degradation, primary production, and nutrient cycling (Kirchman et al. 2003, Yamada et al. 2006, Rigonato et al. 2013). Although some core genera were ubiquitously distributed across all conditions, others varied in relative abundance or were preferentially enriched under specific environmental contexts, suggesting that environmental selection modulate abundance rather than presence or absence.

Urban microbial mats exhibited a higher representation of unique and rare taxa; a pattern frequently observed in disturbed ecosystems where environmental variability or nutrient inputs generate novel ecological niches. Rare microbial lineages may therefore act as sensitive indicators of environmental change (Shade et al. 2012).

Several taxa enriched or exclusively detected in urban mats, including *Ferrimicrobium* spp., *Thermonospora* spp., *Alcanivorax* spp., and *Serratia* spp, have previously been associated with contaminated, urbanized, or otherwise disturbed environments (Yakimov et al. 1998, Johnson et al. 2009, Wei et al. 2014, Alvarez et al. 2017, Behera et al. 2017, Pereira et al. 2023). The enrichment of these metabolically versatile and stress-tolerant taxa suggest that urban conditions may favor microbial lineages capable of adapting to fluctuating or disturbed habitats. However, because specific contaminants were not measured, these patterns should be interpreted as associations with anthropogenic influence rather than direct evidence of pollutant exposure. Future studies incorporating direct measurements of environmental contaminants, such as hydrocarbons or heavy metals, will be crucial to clarify the relationship between pollution and microbial community structure in mangrove sediments.

In contrast, Green Sulfur Bacteria (GSB) such as *Prosthecochloris* spp. and *Chlorobaculum* spp., were detected only in protected and natural sites, where they may benefit from stable, stratified microenvironments. These anoxygenic phototrophs require sulfur-rich, anaerobic conditions with sufficient light penetration and are known to be sensitive to oxygen exposure and sediment disturbance (Roeselers et al. 2007, Bedard et al. 2023). Their absence from urban mats may therefore reflect disruption of the microenvironmental stratification required for their persistence. Although dissolved oxygen measurements showed inconsistent associations with GSB abundance across sites, this pattern further supports the idea that bulk physicochemical measurements do not capture fine-scale microhabitat heterogeneity.

### Beggiatoaceae lineage dynamics across environmental conditions

Members of the Beggiatoaceae family were present across all sampling sites, confirming their central structural and ecological role in the formation of microbial mats in mangrove sediments. They contribute to mat development both by physically shaping the mat matrix and by providing ecological niches that support diverse microbial communities. Despite their ecological significance and likely major contribution to mat biomass, Beggiatoaceae were not among the most abundant taxa in the 16S dataset. This apparent discrepancy may reflect both biological and methodological factors, as large filamentous bacteria can contribute disproportionately to mat biomass while representing a smaller fraction of total cell counts and may be underrepresented in amplicon-based datasets due to DNA extraction and primer biases. Therefore, relative abundance estimates should not be interpreted as direct indicators of ecological dominance in terms of biomass or structural influence.

The structural importance of Beggiatoaceae despite their moderate relative abundance is consistent with findings from other sulfur-rich ecosystems, where sulfur-oxidizing taxa strongly influenced microbial mat architecture and occupy distinct niches structured by micro-scale environmental gradients (Crépeau et al. 2011). Likewise, recent metabarcoding studies in sulfur-rich sinkholes have shown that these communities can be highly specialized and geographically structured (Fray et al. 2024), supporting the idea that local environmental conditions shape the taxonomic assembly of microbial mats.

Clear shifts in Beggiatoaceae lineage composition were observed among sites at the genus level: *Ca*. Marithioploca spp. were most abundant in natural sites, whereas *Ca*. Maribeggiatoa spp., predominated in urban environments, suggesting lineage-level ecological differentiation along the disturbance gradient. Similar niche differentiation among Beggiatoaceae taxa has been reported in other sulfur-rich marine systems, where closely related lineages occupy distinct redox and sulfide niche depending on local environmental conditions (Mußmann et al. 2007, Gutierrez et al. 2008, Grünke et al. 2011, Teske and Salman 2014).

Nutrient exchange between Beggiatoaceae and sulfate-reducing bacteria has been proposed in sulfur-rich ecosystems (Macalady et al. 2008, Teske and Salman 2014, Sharrar et al. 2017), although such interactions remain to be investigated in mangrove environments. In addition, sulfur associated genera such as *Sulfurovum* spp. and *Sulfurimonas* spp., were more abundant in natural and urban sites. These genera are commonly reported in sulfur-rich and redox stratified environments (Aranda et al. 2015, Moulana et al. 2020, Nosalova et al. 2023). Members of the genus *Sulfurovum* spp. have been reported as dominant components of Beggiatoaceae-associated communities in deep-sea mats (Flood et al. 2021). Although direct ecological interactions cannot be inferred from amplicon data alone, these patterns suggest that white microbial mats of Beggiatoaceae occur within a broader sulfur-associated microbial consortium.

Altogether, these observations highlight the taxonomic plasticity and environmental sensitivity of dominant sulfur-oxidizing microbial guilds. The presence or turnover of specific sulfur-associated taxa may serve as indicators of ecological change in mangrove ecosystems. Future genomic and functional approaches will be essential to better understand the metabolic roles, ecological interactions and resilience mechanisms of microbial mats under increasing anthropogenic pressure.

## Supplementary Material

xtag034_Supplemental_Files

## References

[bib1] Albuquerque JPd, Keim CN, Lins U. Comparative analysis of beggiatoa from hypersaline and marine environments. Micron. 2010;41:507–17. 10.1016/j.micron.2010.01.009 20207153

[bib2] Allison SD, Martiny JBH. Resistance, resilience, and redundancy in microbial communities. Proc Natl Acad Sci USA. 2008;105:11512–9. 10.1073/pnas.0801925105 18695234 PMC2556421

[bib3] Alvarez A, Saez JM, Davila Costa JS et al. Actinobacteria: current research and perspectives for bioremediation of pesticides and heavy metals. Chemosphere. 2017;166:41–62. 10.1016/j.chemosphere.2016.09.070 27684437

[bib4] Alzubaidy H, Essack M, Malas TB et al. Rhizosphere microbiome metagenomics of gray mangroves (Avicennia marina) in the Red Sea. Gene. 2016;576:626–36. 10.1016/j.gene.2015.10.032 26475934

[bib5] Andreote FD, Jiménez DJ, Chaves D et al. The microbiome of Brazilian mangrove sediments as revealed by metagenomics. PLoS One. 2012;7:e38600. 10.1371/journal.pone.0038600 22737213 PMC3380894

[bib6] Aranda CP, Valenzuela C, Matamala Y et al. Sulphur-cycling bacteria and ciliated protozoans in a Beggiatoaceae mat covering organically enriched sediments beneath a salmon farm in a southern Chilean fjord. Mar Pollut Bull. 2015;100:270–8. 10.1016/j.marpolbul.2015.08.040 26359117

[bib7] Asari N, Suratman MN, Mohd Ayob NA et al. Mangrove as a natural barrier to environmental risks and coastal protection. In: Mangroves: Ecology, Biodiversity and Management. n.p.: Springer Nature, 2021, 305–22. 10.1007/978-981-16-2494-0_13

[bib8] Baumgartner LK, Reid RP, Dupraz C et al. Sulfate reducing bacteria in microbial mats: changing paradigms, new discoveries. Sediment Geol. 2006;185:131–45. 10.1016/j.sedgeo.2005.12.008

[bib9] Bedard DL, Slyke GV, Nübel U et al. Geographic and ecological diversity of green sulfur bacteria in hot spring mat communities. Microorganisms. 2023;11:2921. 10.3390/microorganisms11122921 38138064 PMC10746008

[bib10] Behera BC, Yadav H, Singh SK et al. Phosphate solubilization and acid phosphatase activity of Serratia sp. isolated from mangrove soil of Mahanadi River Delta, Odisha, India. J Genetic Engineering Biotechnol. 2017;15:169–78. 10.1016/j.jgeb.2017.01.003 PMC6296638 30647653

[bib11] Bengtsson-Palme J, Hartmann M, Eriksson KM et al. metaxa2: improved identification and taxonomic classification of small and large subunit rRNA in metagenomic data. Mol Ecol Resour. 2015;15:1403–14. 10.1111/1755-0998.12399 25732605

[bib12] Bernard C, Fenchel T. Mats of colourless sulphur bacteria. II. Structure, composition of biota and successional patterns. Mar Ecol Prog Ser. 1995;128:171–9. 10.3354/meps128171

[bib13] Bouchez A, Pascault N, Chardon C et al. Mangrove microbial diversity and the impact of trophic contamination. Mar Pollut Bull. 2013;66:39–46. 10.1016/j.marpolbul.2012.11.015 23218774

[bib14] Buck KR, Barry JP, Hallam SJ. Thioploca spp. Sheaths as niches for bacterial and protistan assemblages. Mar Ecol. 2014;35:395–400. 10.1111/maec.12076

[bib15] Cadena S, Teutli-Hernández C, Herrera-Silveira JA et al. Microbial diversity of coastal microbial mats formations in karstic habitats from the Yucatan Peninsula. PLoS One. 2025;20:e0325200. 10.1371/journal.pone.0325200 40460360 PMC12133189

[bib16] Chen H, Boutros PC. VennDiagram: Generate High-Resolution Venn and Euler Plot, 1.7.3. Preprint, 2022.

[bib17] Crépeau V, Cambon Bonavita MA, Lesongeur F et al. Diversity and function in microbial mats from the Lucky Strike hydrothermal vent field. FEMS Microbiol Ecol. 2011;76:524–40. 10.1111/j.1574-6941.2011.01070.x 21348883

[bib18] Dray S, Dufour AB. The ade4 Package: implementing the duality diagram for ecologists. J Stat Soft. 2007;22:1–20. 10.18637/jss.v022.i04

[bib19] Dunker R, Røy H, Kamp A et al. Motility patterns of filamentous sulfur bacteria, beggiatoa spp. FEMS Microbiol Ecol. 2011;77:176–85. 10.1111/j.1574-6941.2011.01099.x 21446951

[bib20] Escobar-Zepeda A, De LAVP, Sanchez-Flores A. The road to metagenomics: from microbiology to DNA sequencing technologies and bioinformatics. Front Genet. 2015;6:348. 10.3389/fgene.2015.00348 26734060 PMC4681832

[bib21] Flood BE, Louw DC, Plas AKVd et al. Giant sulfur bacteria (Beggiatoaceae) from sediments underlying the Benguela upwelling system host diverse microbiomes. PLoS One. 2021;16:1–31. 10.1371/journal.pone.0258124 PMC8612568 34818329

[bib22] Fray D, McGovern CA, Casamatta DA et al. Metabarcoding reveals unique microbial mat communities and evidence of biogeographic influence in low-oxygen, high-sulfur sinkholes and springs. Ecol Evol. 2024;14:e11162. 10.1002/ece3.11162 38529029 PMC10961586

[bib23] Gerdes G. What are microbial mats? In: Seckbach J, Oren A (eds.), Microbial Mats. Cellular Origin, Life in Extreme Habitats and Astrobiology. Vol. 14, Dordrecht: Springer, 2010, 3–25. 10.1007/978-90-481-3799-2_1

[bib24] Ghose M, Parab AS, Manohar CS et al. Unraveling the role of bacterial communities in mangrove habitats under the urban influence, using a next-generation sequencing approach. J Sea Res. 2024;198:102469. 10.1016/j.seares.2024.102469

[bib25] Gloor GB, Macklaim JM, Fernandes AD. Displaying variation in large datasets: plotting a visual summary of effect sizes. J Comput Graph Statist. 2016;25:971–9. 10.1080/10618600.2015.1131161

[bib26] Gómez-Acata ES, Teutli C, Falcón LI et al. Sediment microbial community structure associated to different ecological types of mangroves in Celestún, a coastal lagoon in the Yucatan Peninsula, Mexico. PeerJ. 2023;11:e14587. 10.7717/peerj.14587 36785710 PMC9921989

[bib27] Grünke S, Felden J, Lichtschlag A et al. Niche differentiation among mat-forming, sulfide-oxidizing bacteria at cold seeps of the Nile Deep Sea Fan (Eastern Mediterranean Sea). Geobiology. 2011;9:330–48. 10.1111/j.1472-4669.2011.00281.x 21535364

[bib28] Guidi-Rontani C, Jean MRN, Gonzalez-Rizzo S et al. Description of new filamentous toxic cyanobacteria (Oscillatoriales) colonizing the sulfidic periphyton mat in marine mangroves. FEMS Microbiol Lett. 2014;359:173–81. 10.1111/1574-6968.12551 25088450

[bib29] Gutierrez D, Enriquez E, Purca S et al. Oxygenation episodes on the continental shelf of central Peru: remote forcing and benthic ecosystem response. Prog Oceanogr. 2008;79:177–89. 10.1016/j.pocean.2008.10.025

[bib30] Jean MRN, Gonzalez-Rizzo S, Gauffre-Autelin P et al. Two new Beggiatoa species inhabiting marine mangrove sediments in the Caribbean. PLoS One. 2015;10:e0117832. 10.1371/journal.pone.0117832 25689402 PMC4331518

[bib31] Johnson DB, Bacelar-Nicolau P, Okibe N et al. Ferrimicrobium acidiphilum gen. nov., sp. nov. And ferrithrix thermotolerans gen. nov., sp. nov.: heterotrophic, iron-oxidizing, extremely acidophilic actinobacteria. Int J Syst Evol Microbiol. 2009;59:1082–9. 10.1099/ijs.0.65409-0 19406797

[bib32] Jörgensen BB, Gallardo VA. Thioploca spp.: filamentous sulfur bacteria with nitrate vacuoles. FEMS Microbiol Ecol. 1999;28:301–13. 10.1016/S0168-6496(98)00122-6

[bib33] K A, Parveen KH, K SV et al. Mangroves in environmental engineering: harnessing the multifunctional potential of nature’s coastal architects for sustainable ecosystem management. Results in Engineering. 2024;21:101765. 10.1016/j.rineng.2024.101765

[bib34] Kirchman DL, Yu L, Cottrell MT. Diversity and abundance of uncultured cytophaga-like bacteria in the Delaware Estuary. Appl Environ Microb. 2003;69:6587–96. 10.1128/AEM.69.11.6587-6596.2003 PMC262274 14602617

[bib35] Klindworth A, Pruesse E, Schweer T et al. Evaluation of general 16S ribosomal RNA gene PCR primers for classical and next-generation sequencing-based diversity studies. Nucleic Acids Res. 2013;41:e1–e1. 10.1093/nar/gks808 22933715 PMC3592464

[bib36] Li Y, Zheng L, Zhang Y et al. Comparative metagenomics study reveals pollution induced changes of microbial genes in mangrove sediments. Sci Rep. 2019;9:5739. 10.1038/s41598-019-42260-4 30952929 PMC6450915

[bib37] Luis P, Saint-Genis G, Vallon L et al. Contrasted ecological niches shape fungal and prokaryotic community structure in mangroves sediments. Environ Microbiol. 2019;21:1407–24. 10.1111/1462-2920.14571 30807675

[bib38] Macalady JL, Dattagupta S, Schaperdoth I et al. Niche differentiation among sulfur-oxidizing bacterial populations in cave waters. ISME Journal. 2008;2:590–601. 10.1038/ismej.2008.25 18356823

[bib39] Magoč T, Salzberg SL. FLASH: fast length adjustment of short reads to improve genome assemblies. Bioinformatics. 2011;27:2957. 10.1093/bioinformatic/btr507 21903629 PMC3198573

[bib40] Maiti SK, Chowdhury A. Effects of anthropogenic pollution on mangrove biodiversity: a review. JEP. 2013;04:1428–34. 10.4236/jep.2013.412163

[bib41] Mazière C, Duran R, Dupuy C et al. Microbial mats as model to decipher climate change effect on microbial communities through a mesocosm study. Front Microbiol. 2023;14:1039658. 10.3389/fmicb.2023.1039658 37396368 PMC10308941

[bib42] McMurdie PJ, Holmes S. Phyloseq: an R package for reproducible interactive analysis and graphics of microbiome census data. PLoS One. 2013;8:e61217. 10.1371/journal.one.0061217 23630581 PMC3632530

[bib43] Moulana A, Anderson RE, Fortunato CS et al. Selection is a significant driver of gene gain and loss in the pangenome of the bacterial genus sulfurovum in geographically distinct deep-sea hydrothermal vents. MSystems. 2020;5:e00673–19. 10.1128/msystems.00673-19 32291353 PMC7159903

[bib44] Mußmann M, Hu FZ, Richter M et al. Insights into the genome of large sulfur bacteria revealed by analysis of single filaments. PLoS Biol. 2007;5:1923–37. 10.1371/journal.pbio.0050230 PMC1951784 17760503

[bib45] Mußmann M, Schulz HN, Strotmann B et al. Phylogeny and distribution of nitrate-storing beggiatoa spp. in coastal marine sediments. Environ Microbiol. 2003;5:523–33. 10.1046/j.1462-2920.2003.00440.x 12755720

[bib46] Nemergut DR, Schmidt SK, Fukami T et al. Patterns and processes of microbial Community assembly. Microbiol Mol Biol Rev. 2013;77:342–56. 10.1128/mmbr.00051-12 24006468 PMC3811611

[bib47] Nosalova L, Mekadim C, Mrazek J et al. Thiothrix and sulfurovum genera dominate bacterial mats in Slovak cold sulfur springs. Environ Microbiomes. 2023;18:72. 10.1186/s40793-023-00527-4 PMC10512639 37730677

[bib48] Pascal PY, Dubois S, Boschker HTS et al. Trophic role of large benthic sulfur bacteria in mangrove sediment. Mar Ecol Prog Ser. 2014;516:127–38. 10.3354/meps11035

[bib49] Pereira ÉJMC, Amorim ÉA, da F et al. Biocontrol potential of Serratia Marcescens (B8) and Bacillus sp. (B13) Isolated from urban mangroves in Raposa. Life. 2023;13:2036. 10.3390/life13102036 37895418 PMC10607943

[bib50] Preisner EC, Fichot EB, Norman RS. Microbial mat compositional and functional sensitivity to environmental disturbance. Front Microbiol. 2016;7:1632. 10.3389/fmicb.2016.01632 27799927 PMC5066559

[bib51] Prieto-Barajas CM, Valencia-Cantero E, Santoyo G. Microbial mat ecosystems: structure types, functional diversity, and biotechnological application. Electron J Biotechnol. 2018;31:48–56. 10.1016/j.ejbt.2017.11.001

[bib52] Quintero IJ, Castillo AM, Mejía LC. Diversity and taxonomy of soil bacterial communities in urban and rural mangrove forests of the Panama Bay. Microorganisms. 2022;10:2191. 10.3390/microorganisms10112191 36363784 PMC9697262

[bib53] Rigonato J, Kent AD, Alvarenga DO et al. Drivers of cyanobacterial diversity and community composition in mangrove soils in south-east Brazil. Environ Microbiol. 2013;15:1103–14. 10.1111/j.1462-2920.2012.02830.x 22816485

[bib54] Roeselers G, Norris TB, Castenholz RW et al. Diversity of phototrophic bacteria in microbial mats from Arctic hot springs (Greenland). Environ Microbiol. 2007;9:26–38. 10.1111/j.1462-2920.2006.01103.x 17227409

[bib55] Salman V, Amann R, Girnth AC et al. A single-cell sequencing approach to the classification of large, vacuolated sulfur bacteria. Syst Appl Microbiol. 2011;34:243–59. 10.1016/j.syapm.2011.02.001 21498017

[bib56] Salman V, Bailey JV, Teske A. Phylogenetic and morphologic complexity of giant sulphur bacteria. Antonie Van Leeuwenhoek. 2013;104:169–86. 10.1007/s10482-013-9952-y 23793621

[bib57] Santos HFD, Cury JC, Carmo FLd et al. Mangrove bacterial diversity and the impact of oil contamination revealed by pyrosequencing: bacterial proxies for oil pollution. PLoS One. 2011;6:e16943. 10.1371/journal.pone.0016943 21399677 PMC3047533

[bib58] Schnetger B, Lehners C. Determination of nitrate plus nitrite in small volume marine water samples using vanadium (III) chloride as a reduction agent. Mar Chem. 2014;160:91–98. 10.1016/j.marchem.2014.01.010

[bib59] Schulz HN, Jørgensen BB. Big bacteria. Annu Rev Microbiol. 2001;55:105–37. 10.1146/annurev.micro.55.1.105 11544351

[bib60] Shade A, Peter H, Allison SD et al. Fundamentals of microbial community resistance and resilience. Front Microbio. 2012;3:417. 10.3389/FMICB.2012.00417 PMC3525951 23267351

[bib61] Sharrar AM, Flood BE, Bailey JV. et al. Novel large sulfur bacteria in the metagenomes of groundwater-fed chemosynthetic microbial mats in the Lake Huron basin. Front Microbiol. 2017;8:791. 10.3389/fmicb.2017.00791 28533768 PMC5421297

[bib62] Solorzano L. Determination of ammonia in natural waters by the phenolhypochlorite method. Limnol Oceanogr. 1969;14:799–801. 10.4319/lo.1969.14.5.0799

[bib63] Song S, Ding Y, Li W et al. Mangrove reforestation provides greater blue carbon benefit than afforestation for mitigating global climate change. Nat Commun. 2023;14:756. 10.1038/s41467-023-36477-1 36765059 PMC9918466

[bib64] Stal LJ. Coastal microbial mats: the physiology of a small-scale ecosystem. S Afr J Bot. 2001;67:399–410. 10.1016/S0254-6299(15)31156-X

[bib65] Su X, Pan W, Song B et al. Parallel-META 2.0: enhanced metagenomic Data analysis with functional annotation, high performance computing and advanced visualization. PLoS One. 2014;9:e89323. 10.1371/journal.one.0089323 24595159 PMC3940597

[bib66] Teske A, Salman V. The family beggiatoaceae. In: Rosenberg E, DeLong EF, Lory S et al. (eds.), The Prokaryotes. n.p. Berlin, Heidelberg: Springer, 2014, 93–134. 10.1007/978-3-642-38922-1_290

[bib67] Ullah R, Yasir M, Khan I et al. Comparative bacterial community analysis in relatively pristine and anthropogenically influenced mangrove ecosystems on the Red Sea. Can J Microbiol. 2017;63:649–60. 10.1139/cjm-2016-0587 28376307

[bib68] Wei R, Oeser T, Then J et al. Functional characterization and structural modeling of synthetic polyester-degrading hydrolases from thermomonospora curvata. AMB Expr. 2014;4:1–10. 10.1186/s13568-014-0044-9 PMC4231364 25405080

[bib69] Wickham H, Averick M, Bryan J et al. Welcome to the Tidyverse. JOSS. 2019;4:1686. 10.21105/joss.01686

[bib70] Wickham H, Chang W, Henry L et al. ggplot2: Elegant Graphics for Data Analysis., 3.4.3. Preprint, New York: Spinger-Verlag 2016. 10.1007/978-3-319-24277-4_9

[bib71] Yakimov MM, Golyshin PN, Lang S et al. Alcanivorax borkumensis gen. nov., sp. nov., a new, hydrocarbon- degrading and surfactant-producing marine bacterium. Int J Syst Bacteriol. 1998;48:339–48. 10.1099/00207713-48-2-339 9731272

[bib72] Yamada T, Sekiguchi Y, Hanada S et al. Anaerolinea thermolimosa sp. nov., levilinea saccharolytica gen. nov., sp. nov. And leptolinea tardivitalis gen. nov., sp. nov., novel filamentous anaerobes, and description of the new classes anaerolineae classis nov. And caldilineae classis nov. in the bacterial phylum Chloroflexi. Int J Syst Evol Microbiol. 2006;56:1331–40. 10.1099/ijs.0.64169-0 16738111

[bib73] Zhou Z, Meng H, Liu Y et al. Stratified bacterial and archaeal community in mangrove and intertidal wetland mudflats revealed by high throughput 16S rRNA gene sequencing. Front Microbiol. 2017;8:2148. 10.3389/fmicb.2017.02148 29163432 PMC5673634

