# Supplementary material for: Revealing the bacterial diversity and variation of white filamentous microbial mats in marine mangroves of Guadeloupe Island in relation to human activities
Source: FEMS Microbes. 2026 Jun 16;7:xtag034. doi: 10.1093/femsmc/xtag034 (PMC13296560; doi:10.1093/femsmc/xtag034)
Supplement: xtag034_Supplemental_Files [file xtag034_supplemental_files.zip › Supplemental_figures_NV_rev_060326.pptx]

## Slide 1
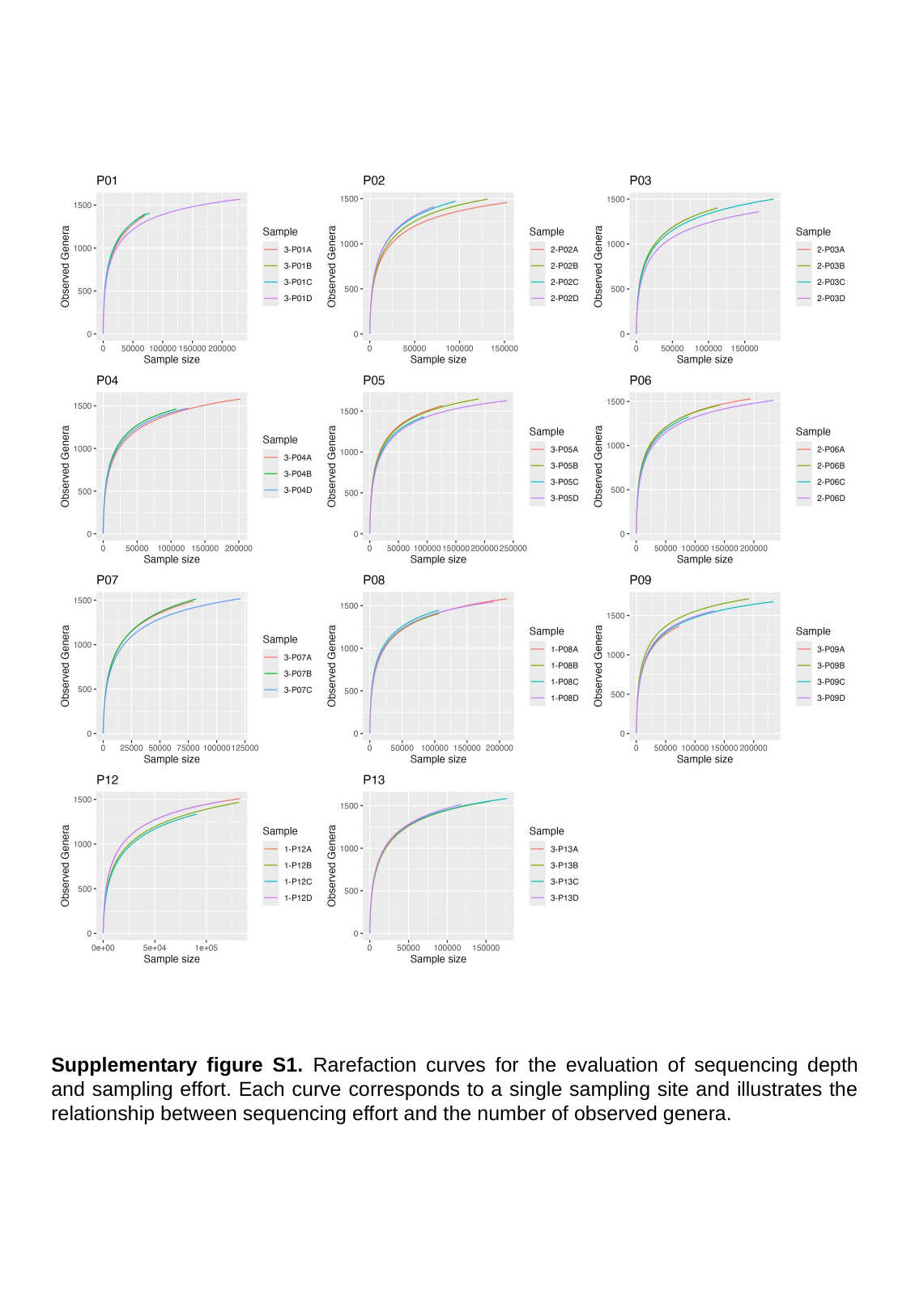

Supplementary figure S1. Rarefaction curves for the evaluation of sequencing depth and sampling effort. Each curve corresponds to a single sampling site and illustrates the relationship between sequencing effort and the number of observed genera.

## Slide 2
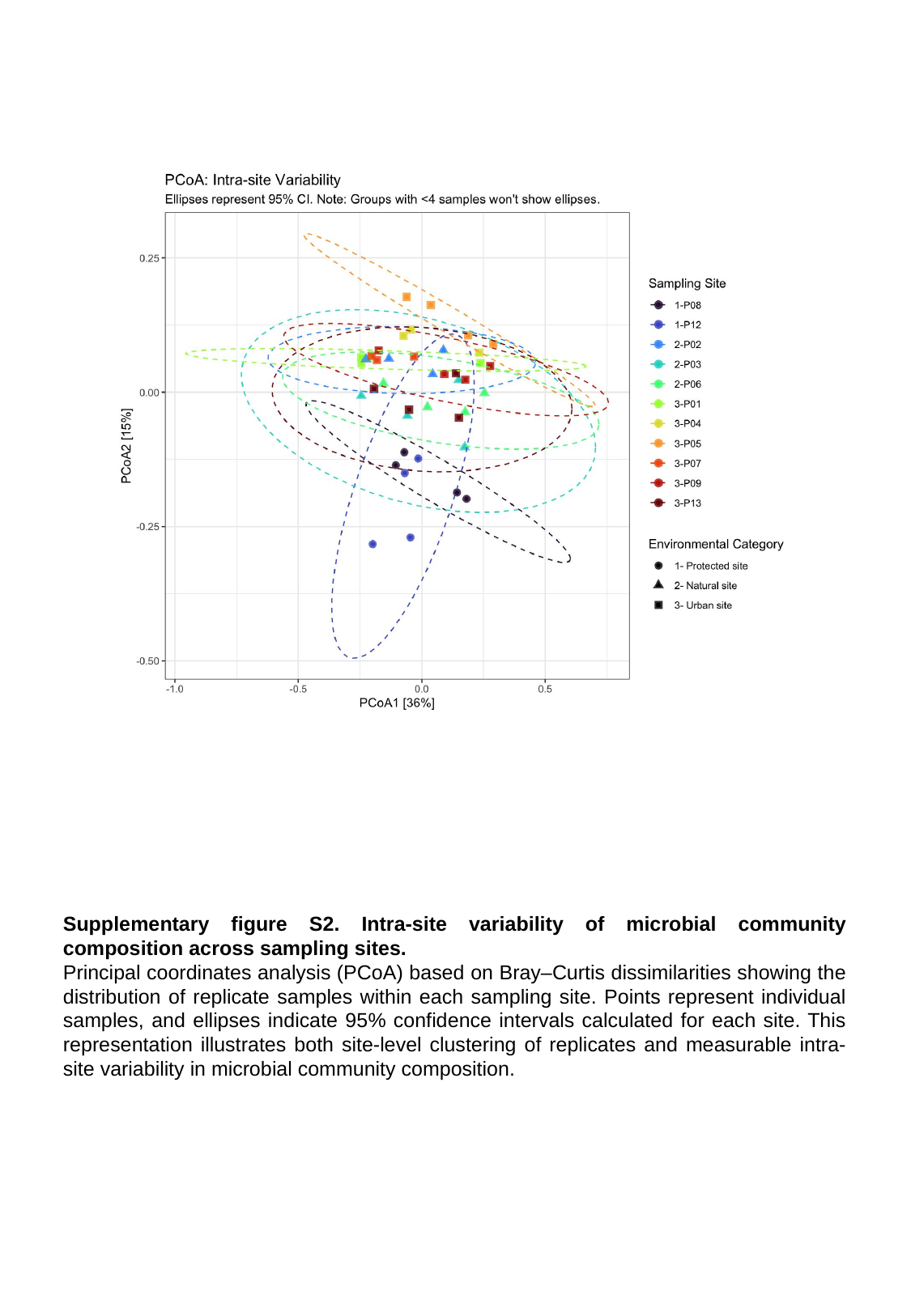

Supplementary figure S2. Intra-site variability of microbial community composition across sampling sites.
Principal coordinates analysis (PCoA) based on Bray–Curtis dissimilarities showing the distribution of replicate samples within each sampling site. Points represent individual samples, and ellipses indicate 95% confidence intervals calculated for each site. This representation illustrates both site-level clustering of replicates and measurable intra-site variability in microbial community composition.

## Slide 3
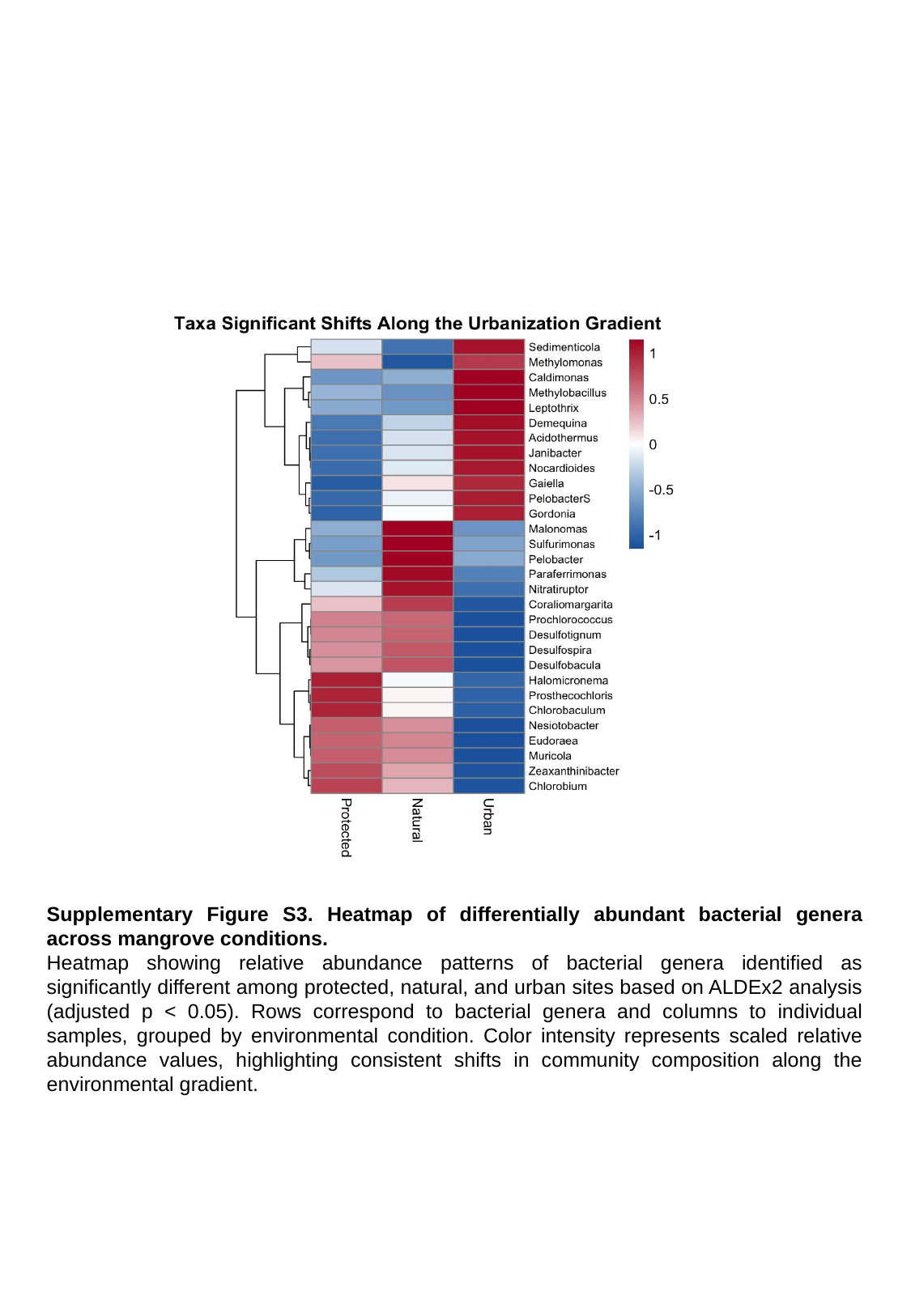

Supplementary Figure S3. Heatmap of differentially abundant bacterial genera across mangrove conditions.
Heatmap showing relative abundance patterns of bacterial genera identified as significantly different among protected, natural, and urban sites based on ALDEx2 analysis (adjusted p < 0.05). Rows correspond to bacterial genera and columns to individual samples, grouped by environmental condition. Color intensity represents scaled relative abundance values, highlighting consistent shifts in community composition along the environmental gradient.

## Slide 4
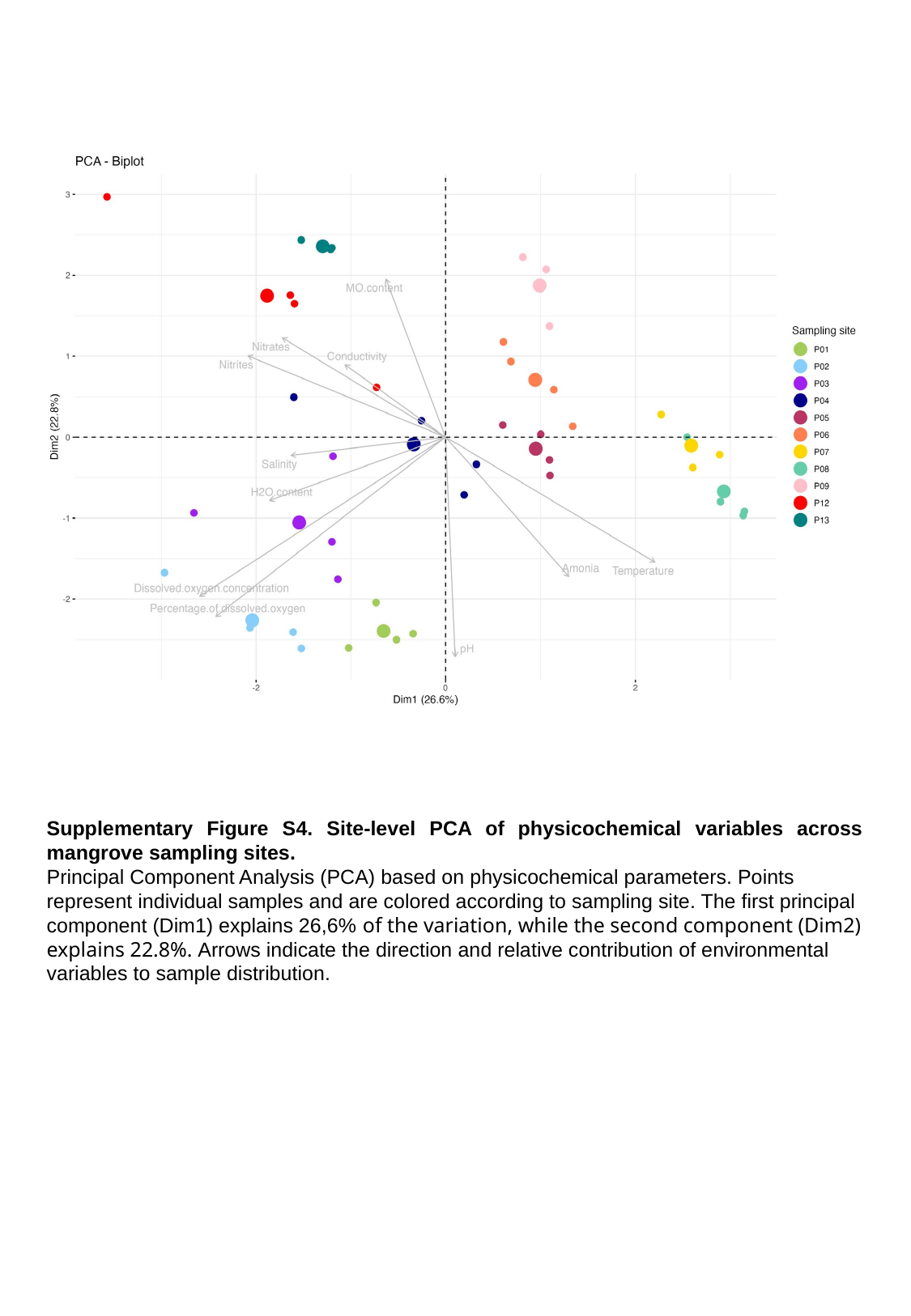

Supplementary Figure S4. Site-level PCA of physicochemical variables across mangrove sampling sites.
Principal Component Analysis (PCA) based on physicochemical parameters. Points represent individual samples and are colored according to sampling site. The first principal component (Dim1) explains 26,6% of the variation, while the second component (Dim2) explains 22.8%. Arrows indicate the direction and relative contribution of environmental variables to sample distribution.
