# Supplementary material for: Revealing the bacterial diversity and variation of white filamentous microbial mats in marine mangroves of Guadeloupe Island in relation to human activities
Source: FEMS Microbes. 2026 Jun 16;7:xtag034. doi: 10.1093/femsmc/xtag034 (PMC13296560; doi:10.1093/femsmc/xtag034)
Supplement: xtag034_Supplemental_Files [file xtag034_supplemental_files.zip › FEMSMC-2025-078.R2 supplementary data - reviews and author response.docx]

**Reviews of manuscript FEMSMC-2025-078**

**Reviewer 1**

**Recommendation: Major Revision**

The manuscript by Martinez-Noriega et al. explores the prokaryotic communities associated with white filamentous microbial mats in mangroves from Guadeloupe. The design of the experiments allows them to explore the communities from 11 different sites from 3 different areas qualified as “protected” (2 sites, 8 samples), “natural” (3 sites, 12 samples), and “urban” (6 sites, 22 samples). Analyses of the presence or absence of taxa at different taxonomic ranks reveal differences in community composition according to site location.

The manuscript by Martinez-Noriega et al. also focuses on a specific bacterial family, the Beggiatoaceae, which are known to contain primarily sulfur-oxidizing autotrophs that are commonly found in dense mats. These bacteria correspond to an interesting family that really deserves more investigations regarding their abundance, diversity, and functional roles in mangroves. While I acknowledge the interest of the work, and the relevance of the topic, I have some major comments regarding statistical analysis and data interpretation. Please find my remarks below.

Introduction

Overall, the Introduction section is interesting. The authors might consider adding a few references that have used metabarcoding approaches to study microbial mats, although not from mangroves. These studies might also help in the Discussion section for the roles of Beggiatoaceae. Among them: (1) Fray et al. [Fray, D., McGovern, C. A., Casamatta, D. A., Biddanda, B. A., & Hamsher, S. E. (2024). Metabarcoding reveals unique microbial mat communities and evidence of biogeographic influence in low-oxygen, high-sulfur sinkholes and springs. Ecology and Evolution, 14, e11162. https://doi.org/10.1002/ece3.11162] use 16S DNA metabarcoding to investigate the composition of white filaments, (2) Crépeau et al. [Valentin Crépeau, Marie-Anne Cambon Bonavita, Françoise Lesongeur, Henintsoa Randrianalivelo, Pierre-Marie Sarradin, Jozée Sarrazin, Anne Godfroy, Diversity and function in microbial mats from the Lucky Strike hydrothermal vent field, FEMS Microbiology Ecology, Volume 76, Issue 3, Pages 524–540, https://doi.org/10.1111/j.1574-6941.2011.01070.x] used DNA metabarcoding approaches.

Materials and Methods

More details on the bioinformatic procedures used to analyze the metabarcoding data would be welcome. The authors refer to the article by Escobar-Zepeda et al. (2018) which is essentially a comparison of different methods. Metataxa2 is essentially a taxonomic classifier, and mentioning the parallel-META pipeline is not sufficient for people who are not using this pipeline to fully understand the different steps.

Results

The authors have data from three to four samples at each site, which is a very interesting design that should be used to address intra-site variability for both the composition of the prokaryotic community and biochemical data. These important analyses are not presented in the manuscript and all samples are treated independently, even if variability in community assembly can be easily seen; for example the Fig. 6 reveal some intra-site heterogeneity in Beggiatoaceae richness and abundance. I suggest that more statistical analyses should be performed to test both differences between “environmental conditions” and differences within environmental areas.

Although the presented NMDS indicates difference in community composition (confirmed by Permanova analyses), I wonder whether the authors could present complementary analyses such as PERMDISP2, MANOVA …. to further demonstrate differences between “environments” knowing that there are differences within replicates (also see above).

The presented figures (Fig. 4 and Fig. 5) which reveal differences in community assembly, are not very demonstrative. Again, statistical information is required to know which taxa are differentially abundant between “compartments”. The authors should use available packages (DESeq2, ALDEx2, ANCOM-BC …).

The analyses of the core microbiome were performed at the genera taxonomic rank. Further analyses could be presented, but at other taxonomic ranks.

For the physicochemical analyzes, the authors should give some information on the variability within sites (replicates). Approaches, such as PERMANOVA with environmental variables or Spearman/Pearson correlations …, should help to compare physicochemical data with 16S taxonomic composition across three “compartments”.

Discussion

Intraspecies metabolic diversity has been demonstrated, and it is known that metabolic functions do not always correlate with 16S taxonomy. Even if this article is already interesting, the authors should be more cautious in the Discussion section.

Lignes 375-382: I recommend that discussing the putative ecological roles based only on class analyses should be performed with more caution.

Ligne 382-387: The authors mention the possibility of co-occurrence between Sulfurovum spp. and Beggiatoaceaeae, which is interesting. I wonder whether the authors could also perform co-occurrence analyses with their own data?

Ligne 507-519: The authors discuss the abundance (“enrichment”, abundance was considerably lower” …) of several taxa without giving information on their relative levels.

**Reviewer 2: Mirna Vazquez**

**Recommendation: Minor Revision**

Major comments

Please clarify whether human-induced environmental changes refers broadly to human presence and physical disturbance (e.g. urban activity, waste, navigation), rather than to specific pollutants, which were not directly measured.

The first part of the Discussion largely reiterates patterns already described in the Results (alpha and beta diversity differences among site categories). The manuscript would benefit from condensing this section and focusing more directly on interpretation and ecological mechanisms rather than restating observed patterns. More generally, the Discussion could be substantially streamlined to reduce redundancy and highlight the key ecological insights more clearly.

Given that the physicochemical PCA suggests clustering at the site level, it would be helpful to clarify whether beta-diversity patterns are primarily driven by environmental condition or by site-specific effects (e.g. by including site as a factor or nested term in PERMANOVA, or by reporting effect sizes).

Consider reporting pairwise PERMANOVA effect sizes (R²) to better contextualize the degree of similarity between natural and urban sites, particularly given their partial overlap in ordination space.

Minor comments

Clarify whether taxa described as absent were below detection thresholds rather than completely absent.

A brief justification of the abundance thresholds used to define core and unique taxa in the Results section would strengthen interpretation.

Given the central ecological role attributed to Beggiatoaceae throughout the manuscript, it may be helpful to briefly discuss the apparent discrepancy between their proposed structural importance and their relatively low relative abundance in the 16S dataset, considering methodological limitations.

Some sections of the Results, particularly at higher taxonomic levels, could be streamlined to reduce descriptive redundancy.

Figure 4 contains a typographical error that should be corrected.

**Associate Editor Recommendation: Major Revision**

Thank you for your patience in waiting for the reviews of your manuscript. As you will see, both reviewers appreciate the concept behind your work but have a number of important recommendations and suggestions. We hope that you are able to address these points in a suitably revised version of your paper.

**EIC Decision: Major Revision**

**Author's Response to Decision Letter for (FEMSMC-2025-078)**

**Reviewer 1.**

**We thank reviewer 1 for the careful reading of our manuscript and for the constructive and insightful comments. Below we addressed the comments and indicated the amends to the manuscript. We also submitted a highlighted version of the manuscript where the chances can be found.**

**Comment 1.**

***Introduction:***

***Overall, the Introduction section is interesting. The authors might consider adding a few references that have used metabarcoding approaches to study microbial mats, although not from mangroves. These studies might also help in the Discussion section for the roles of Beggiatoaceae. Among them: (1) Fray et al. [Fray, D., McGovern, C. A., Casamatta, D. A., Biddanda, B. A., & Hamsher, S. E. (2024). Metabarcoding reveals unique microbial mat communities and evidence of biogeographic influence in low-oxygen, high-sulfur sinkholes and springs. Ecology and Evolution, 14, e11162.*** [***https://doi.org/10.1002/ece3.11162***](https://doi.org/10.1002/ece3.11162)***] use 16S DNA metabarcoding to investigate the composition of white filaments, (2) Crépeau et al. [Valentin Crépeau, Marie-Anne Cambon Bonavita, Françoise Lesongeur, Henintsoa Randrianalivelo, Pierre-Marie Sarradin, Jozée Sarrazin, Anne Godfroy, Diversity and function in microbial mats from the Lucky Strike hydrothermal vent field, FEMS Microbiology Ecology, Volume 76, Issue 3, Pages 524–540,*** [***https://doi.org/10.1111/j.1574-6941.2011.01070.x***](https://doi.org/10.1111/j.1574-6941.2011.01070.x)***] used DNA metabarcoding approaches.***

**Response: We thank the reviewer for this helpful suggestion. We have revised the Introduction to include additional metabarcoding studies on microbial mats in sulfur-rich environments (Fray et al., 2024; Crépeau et al., 2011). These references broaden the methodological context for our 16S-based characterization and strengthen the rationale for applying metabarcoding to white filamentous mats in mangrove sediments.**

**We have also incorporated these references into the Discussion section to contextualize our results.**

**Comment 2.**

***Materials and Methods***

***More details on the bioinformatic procedures used to analyze the metabarcoding data would be welcome. The authors refer to the article by Escobar-Zepeda et al. (2018) which is essentially a comparison of different methods. Metataxa2 is essentially a taxonomic classifier, and mentioning the parallel-META pipeline is not sufficient for people who are not using this pipeline to fully understand the different steps.***

**Response: We agree with the reviewer that the description of the bioinformatic workflow required additional detail. We have expanded the Material and Methods section to provide a better description of the different steps used to process the metabarcoding data and instead of the Escobar-Zepeda et al (2018) reference, we included a github link (**[**https://github.com/Ales-ibt/Metagenomic-benchmark**](https://github.com/Ales-ibt/Metagenomic-benchmark)**) where a step-by-step explanation can be found. These additions aim to make the analytical workflow more transparent and reproductible for readers who are not familiar with this pipeline.**

**Comment 3.**

***Results***

***The authors have data from three to four samples at each site, which is a very interesting design that should be used to address intra-site variability for both community composition and biochemical data. These important analyses are not presented, and all samples are treated independently even if variability in community assembly can be easily seen; for example, the Fig. 6 reveal some intra-site heterogeneity in Beggiatoaceae richness and abundance. I suggest that more statistical analyses should be performed to test both differences between “environmental conditions” and differences within environmental areas.***

**Response: We thank the reviewer for highlighting the importance of site-level replication in our sampling design. To explicitly address intra-site variability, we performed a PERMDISP analysis to test for homogeneity of multivariate dispersions among sites. No significant differences in dispersion were detected (ANOVA, F = 0.423, p = 0.924), indicating that intra-site variability is consistent across the study. We also added a PCoA representation with 95% confidence intervals per site, which illustrates that replicate samples cluster at the site level while still exhibiting measurable intra-site heterogeneity. Together, these results demonstrate that the observed differences in community composition are not driven by unequal dispersion among sites and support the robustness of the PERMANOVA and NMDS interpretations.**

**In addition, within-site variability in physicochemical parameters was evaluated using replicate measurements summarized in Supplementary Table S4 and visualized through a site-level PCA (Supplementary Figure S4).**

**Supplementary figure 2. Intra-site variability of microbial community composition across sampling sites.** Principal coordinates analysis (PCoA) based on Bray–Curtis dissimilarities showing the distribution of replicate samples within each sampling site. Points represent individual samples, and ellipses indicate 95% confidence intervals calculated for each site. This representation illustrates both site-level clustering of replicates and measurable intra-site variability in microbial community composition.

**In addition, we have revised the Discussion section to better explain the variability in Beggiatoaceae richness and relative abundance among replicates, as well as potential methodological limitations associated with the detection of filamentous sulfur-oxidizing bacteria. We emphasize that relative patterns, rather than absolute abundance values, provide the most informative basis for interpretation in this study.**

**Comment 4.**

***Results***

***Although the presented NMDS indicates difference in community composition (confirmed by Permanova analyses), I wonder whether the authors could present complementary analyses such as PERMDISP2, MANOVA …. to further demonstrate differences between “environments” knowing that there are differences within replicates (also see above).***

**Response: We agree with the reviewer that complementary analyses are essential to support NMDS and PERMANOVA interpretations. To address this point, we performed a PERMDISP analysis to test for homogeneity of multivariate dispersions among sites. No significant differences in dispersion were detected (ANOVA, F = 0.423, p = 0.924), indicating that the assumptions of PERMANOVA were met. These results confirm that the observed NMDS separation among environmental conditions reflects genuine differences in community composition rather than differences in within-group dispersion**

**Comment 5.**

**Results**

***The presented figures (Fig. 4 and Fig. 5) which reveal differences in community assembly are not very demonstrative. Again, statistical information is required to know which taxa are differentially abundant between compartments.***

**Response: We agree with the reviewer that descriptive taxonomic profiles alone do not provide statistical support for identifying differentially abundant taxa. To address this point, we performed a differential abundance analysis using ALDEx2, a compositionality-aware approach suitable for 16S rRNA gene data. This analysis allowed us to statistically identify taxa that differ significantly among environmental conditions using a Kruskal–Wallis test with Benjamini–Hochberg correction. The results of this analysis are now reported in the Results section and illustrated using complementary visualizations (bubble plot and heatmap), which support and extend the descriptive patterns shown in Figures 4 and 5.**

**Figure 6. Differentially abundant bacterial genera across mangrove environmental conditions. Bubble plot summarizing the results of the differential abundance analysis performed using ALDEx2 at the genus level. Only genera showing significant differences among protected, natural, and urban sites (Kruskal–Wallis test with Benjamini–Hochberg correction, adjusted p < 0.05) are displayed. Bubble size reflects the effect size, while color intensity represents the strength of the statistical signal. This analysis provides statistical support for taxa associated with the environmental gradient across mangrove conditions.**

**Supplementary Figure S3. Heatmap of differentially abundant bacterial genera across mangrove conditions. Heatmap showing relative abundance patterns of bacterial genera identified as significantly different among protected, natural, and urban sites based on ALDEx2 analysis (adjusted p < 0.05). Rows correspond to bacterial genera and columns to individual samples, grouped by environmental condition. Color intensity represents scaled relative abundance values, highlighting consistent shifts in community composition along the environmental gradient.**

**Comment 6.**

**Results**

***The analyses of the core microbiome were performed at the genera taxonomic rank. Further analyses could be presented, but at other taxonomic ranks.***

**Response: We thank the reviewer for this suggestion. We carefully evaluated the possibility of extending the core microbiome analysis to higher taxonomic ranks. However, for this dataset, genus-level classification provided the most consistent and ecologically interpretable resolution, whereas family-level assignments were affected by database-dependent inconsistencies and unresolved classifications for several taxa. We therefore retained the genus level for core microbiome analyses and clarified this rationale in the revised manuscript.**

**Comment 7**

**Results**

***For the physicochemical analyzes, the authors should give some information on the variability within sites (replicates). Approaches, such as PERMANOVA with environmental variables or Spearman/Pearson correlations …, should help to compare physicochemical data with 16S taxonomic composition across three “compartments”.***

**Response: We thank the reviewer for this valuable suggestion. In the revised manuscript, we clarify within-site variability of physicochemical parameters using replicate measurements summarized in Supplementary Table 4 and explored through principal component analyses. In addition, we included a site-level PCA to visually illustrate environmental heterogeneity among sampling sites, including within the same environmental category (Supplementary Fig. 4).**

**Supplementary Figure S4. Site-level PCA of physicochemical variables across mangrove sampling sites. Principal Component Analysis (PCA) based on physicochemical parameters. Points represent individual samples and are colored according to sampling site. The first principal component (Dim1) explains 26,6% of the variation, while the second component (Dim2) explains 22.8%. Arrows indicate the direction and relative contribution of environmental variables to sample distribution.**

**Comment 8**

***Discussion***

***Intraspecies metabolic diversity has been demonstrated, and it is known that metabolic functions do not always correlate with 16S taxonomy. Even if this article is already interesting, the authors should be more cautious in the Discussion section.***

***Lignes 375-382: I recommend that discussing the putative ecological roles based only on class analyses should be performed with more caution.***

**Response: We thank the reviewer for this comment. We have revised the Discussion to adopt a more cautious interpretation of putative ecological roles inferred from taxonomic data, explicitly acknowledging the limitations of 16S rRNA gene-based analyses for functional inference.**

***Ligne 382-387: The authors mention the possibility of co-occurrence between Sulfurovum spp. and Beggiatoaceaeae, which is interesting. I wonder whether the authors could also perform co-occurrence analyses with their own data ?***

**Response: We thank the reviewer for this insightful comment. We agree that co-occurrence analyses would be valuable to further investigate potential interactions between *Sulfurovum* spp. and Beggiatoaceae. However, in the present study, our interpretations are based on taxonomic distributions and relative abundance patterns, and we did not perform formal co-occurrence analyses. We have revised the Discussion to clarify this limitation and to explicitly state that testing putative interactions between these taxa will require dedicated co-occurrence or network-based analyses in future work.**

***Ligne 507-519: The authors discuss the abundance (“enrichment”, abundance was considerably lower” …) of several taxa without giving information on their relative levels.***

**Response: We thank the reviewer for this comment. We have revised the Discussion to adopt more cautious language when referring to taxon abundance patterns, avoiding terms such as “enrichment” and clarifying that observed differences reflect relative abundance trends rather than quantified effect sizes.**

**Reviewer 2.**

**We thank reviewer 2 for the careful reading of our manuscript and for the constructive comments to improve the manuscript. We also submitted a highlighted version of the manuscript where the chances can be found.**

**Comments to the Author**

**Major comments**

**Comment 1**

***Please clarify whether human-induced environmental changes refers broadly to human presence and physical disturbance (e.g. urban activity, waste, navigation), rather than to specific pollutants, which were not directly measured.***

**Response: We thank the reviewer for this important clarification. As described in Methods section, the term “urban sites” refers to mangrove areas influenced by human presence and activities such as urban infrastructure, navigation, and nearby industrial development. As no specific chemical pollutants were directly measured in this study, the environmental categorization reflects differences in overall anthropogenic influence rather than quantified contaminant levels. This clarification has now been added to the Materials and Methods section.**

**Comment 2**

***The first part of the Discussion largely reiterates patterns already described in the Results (alpha and beta diversity differences among site categories). The manuscript would benefit from condensing this section and focusing more directly on interpretation and ecological mechanisms rather than restating observed patterns. More generally, the Discussion could be substantially streamlined to reduce redundancy and highlight the key ecological insights more clearly.***

**Response: We thank the reviewer for this helpful suggestion. We have revised the first part of the Discussion to reduce descriptive repetition of alpha- and beta-diversity patterns already presented in the Results and to focus more directly on ecological interpretation and underlying mechanisms. Redundant descriptions were condensed, and emphasis was placed on the ecological significance of rare taxa, community stability, and disturbance-related processes.**

**Comment 3**

***Given that the physicochemical PCA suggests clustering at the site level, it would be helpful to clarify whether beta-diversity patterns are primarily driven by environmental condition or by site-specific effects (e.g. by including site as a factor or nested term in PERMANOVA, or by reporting effect sizes).***

***Consider reporting pairwise PERMANOVA effect sizes (R²) to better contextualize the degree of similarity between natural and urban sites, particularly given their partial overlap in ordination space.***

**Response: We appreciate the reviewer’s careful observation regarding the physicochemical PCA and the potential for site-specific effects. We agree with the reviewer that complementary analyses are essential to support NMDS and PERMANOVA interpretations. To address this, we have performed additional analyses to clarify if the beta-diversity patterns are driven by environmental conditions or site-specific effects.**

**We implemented permutation constraints using “site” as a blocking factor to account for localized variance. Despite the hierarchical structure of the sampling design, the global model indicated that environmental condition explains a significant proportion of the variance in community composition (R² = 0.173, p < 0.05).**

**Furthermore, we report pairwise PERMANOVA effect sizes (R²) to contextualize differences among conditions. The comparison between Urban and Natural sites remained statistically significant (p = 0.032) with an effect size of R² = 0.076, whereas the contrast between Urban and Protected sites showed a higher explained variance (R² = 0.165, p = 0.002). These results indicate that environmental categories contribute to structuring beta-diversity patterns beyond site-level variability. In the revised manuscript, we also clarify within-site variability of physicochemical parameters using replicate measurements summarized in Supplementary Table 4 and explored through principal component analyses. In addition, we included a site-level PCA to visually illustrate environmental heterogeneity among sampling sites, including within the same environmental category (Supplementary Fig. 4).**

**Minor comments**

**Comment 1**

***Clarify whether taxa described as absent were below detection thresholds rather than completely absent.***

**Response: We thank the reviewer for this clarification. We have now clarified in the bioinformatic analysis section that taxa described as absent refer to absence in the sequencing dataset under the applied filtering thresholds and may reflect detection limits of the 16S rRNA gene amplicon approach rather than true ecological absence. This clarification has been added to Methods section of the revised manuscript.**

**Comment 2**

***A brief justification of the abundance thresholds used to define core and unique taxa in the Results section would strengthen interpretation.***

**Response:**

**We thank the reviewer for this suggestion. We have now clarified in the material and methods section the rationale behind the relative abundance thresholds use to define core and unique taxa. Specifically, we explain that the selected cut-off of 0,1% were applied to minimize the influence of sporadic-extremely low-abundance taxa while retaining ecologically meaningful genera for robust core microbiome characterization.**

**2.1 (Comment 3)**

***Given the central ecological role attributed to Beggiatoaceae throughout the manuscript, it may be helpful to briefly discuss the apparent discrepancy between their proposed structural importance and their relatively low relative abundance in the 16S dataset, considering methodological limitations.***

**Response:**

**We thank the reviewer for this important observation. We have revised the Discussion to clarify the apparent discrepancy between the structural importance of Beggiatoaceae in microbial mats and their moderate relative abundance in the 16S rRNA dataset. We now discuss both biological factors (e.g., high biomass associated with filamentous morphology versus cell counts) and potential methodological biases inherent to amplicon-based approaches that may lead to underrepresentation of filamentous sulfur-oxidizing bacteria.**

**2.2 (Comment 4)**

***Some sections of the Results, particularly at higher taxonomic levels, could be streamlined to reduce descriptive redundancy.***

**Response: We have streamlined the Results section to reduce descriptive redundancy and focus on the most relevant compositional patterns while referring readers to the figures for detailed abundance values.**

**2.3 (Comment 5)**

***Figure 4 contains a typographical error that should be corrected.***

**Response: We thank the reviewer for noting this typographical issue. Figure 4 has been carefully revised in the updated version of the manuscript.**

**Reviews of manuscript FEMSMC-2025-078.R1**

**Reviewer 1**

**Recommendation: Minor Revision**

Dear Authors,

Thank you for taking into account our comments and remarks. However, the length of the revised manuscript is too long. Indeed, the additional information and analyses that were included in the manuscript essentially consisted of additions to the text with no or almost no rewriting of certain sections. In particular, the discussion needs to be refocused on key ecological perspectives (as indicated by an arbitrator). I recommend additional rewriting efforts.

**Reviewer 2: Mirna Vazquez**

**Recommendation: Accept**

The authors have responded clearly and satisfactorily to the comments, and the revisions have improved the manuscript, particularly in clarifying conceptual points and strengthening the interpretation of the results. The main concerns raised during review have been adequately addressed, and the manuscript is now clearer and more robust overall. I appreciate the authors’ careful and constructive engagement with the suggestions.

**Associate Editor Recommendation: Minor Revision**

Many thanks for your thorough revisions. The manuscript has now been seen by two reviewers again. One of them provides the helpful advice to make a final effort to streamline the text further, in particular the Discussion section. We would like to give you an opportunity to do so to improve the readability of the paper. After this revision, I expect that we should be able to move forward to publication without further re-reviews.

**EIC Decision: Minor Revision**

**Author's Response to Decision Letter for (FEMSMC-2025-078.R1)**

Response to Reviewer 1

Reviewer comment:

The length of the revised manuscript is too long. Indeed, the additional information and analyses that were included in the manuscript essentially consisted of additions to the text with no or almost no rewriting of certain sections. In particular, the discussion needs to be refocused on key ecological perspectives. I recommend additional rewriting efforts.

Response:

Thank you for this constructive feedback. In response, we substantially revised the manuscript in different sections but streamlined the Discussion one to improve readability and better emphasize the key ecological perspectives emerging from our findings.

In particular, the Discussion section has now three integrative thematic sections focused on: (i) diversity patterns and environmental structuring of microbial mats, (ii) core microbiome dynamics and taxonomic shifts associated with disturbance, and (iii) Beggiatoaceae lineage dynamics and sulfur-associated guild restructuring across environmental conditions.

During this revision, we removed redundant descriptive text, consolidated overlapping interpretations, and reduced sections that were overly detailed or result-focused. We also strengthened the ecological framing by emphasizing disturbance-associated community restructuring, niche differentiation among dominant sulfur-oxidizing lineages, and the broader implications of microbial mat plasticity in mangrove ecosystems.

We believe these revisions have substantially improved the clarity, conciseness, and ecological focus of the manuscript.

**Reviews of manuscript FEMSMC-2025-078.R2**

**Associate Editor Recommendation: Accept**

Thank you for addressing the final recommendations of the reviewer. The manuscript looks improved and suitable for publication in FEMS Microbes.

**EIC Decision: Accept**
